# Semi-automated Detection of the Timing of Respiratory Muscle Activity: Validation and First Application

**DOI:** 10.3389/fphys.2021.794598

**Published:** 2022-01-03

**Authors:** Antenor Rodrigues, Luc Janssens, Daniel Langer, Umi Matsumura, Dmitry Rozenberg, Laurent Brochard, W. Darlene Reid

**Affiliations:** ^1^Department of Critical Care, St. Michael's Hospital, Toronto, ON, Canada; ^2^Department of Electrical Engineering, Faculty of Engineering Technology, Katholieke Universiteit Leuven, Leuven, Belgium; ^3^Department of Rehabilitation Sciences, Faculty of Movement and Rehabilitation Sciences, Research Group for Rehabilitation in Internal Disorders, Katholieke Universiteit Leuven, Leuven, Belgium; ^4^Respiratory Rehabilitation and Respiratory Division, University Hospital Leuven, Leuven, Belgium; ^5^Department of Physiotherapy, Nagasaki University, Nagasaki, Japan; ^6^Division of Respirology, Temerty Faculty of Medicine, University of Toronto, University Health Network, Toronto, ON, Canada; ^7^Toronto General Hospital Research Institute, Toronto, ON, Canada; ^8^Interdepartmental Division of Critical Care Medicine, University of Toronto, Toronto, ON, Canada; ^9^Keenan Research Centre, Li Ka Shing Knowledge Institute, St. Michael's Hospital, Toronto, ON, Canada; ^10^Department of Physical Therapy, University of Toronto, Toronto, ON, Canada; ^11^KITE, Toronto Rehabilitation Institute, University Health Network, Toronto, ON, Canada

**Keywords:** respiratory muscles, ventilatory muscles, electromyography, surface electromyography, neck muscles

## Abstract

**Background:** Respiratory muscle electromyography (EMG) can identify whether a muscle is activated, its activation amplitude, and timing. Most studies have focused on the activation amplitude, while differences in timing and duration of activity have been less investigated. Detection of the timing of respiratory muscle activity is typically based on the visual inspection of the EMG signal. This method is time-consuming and prone to subjective interpretation.

**Aims:** Our main objective was to develop and validate a method to assess the respective timing of different respiratory muscle activity in an objective and semi-automated manner.

**Method:** Seven healthy adults performed an inspiratory threshold loading (ITL) test at 50% of their maximum inspiratory pressure until task failure. Surface EMG recordings of the costal diaphragm/intercostals, scalene, parasternal intercostals, and sternocleidomastoid were obtained during ITL. We developed a semi-automated algorithm to detect the onset (EMG, onset) and offset (EMG, offset) of each muscle’s EMG activity breath-by-breath with millisecond accuracy and compared its performance with manual evaluations from two independent assessors. For each muscle, the Intraclass Coefficient correlation (ICC) of the EMG, onset detection was determined between the two assessors and between the algorithm and each assessor. Additionally, we explored muscle differences in the EMG, onset, and EMG, offset timing, and duration of activity throughout the ITL.

**Results:** More than 2000 EMG, onset s were analyzed for algorithm validation. ICCs ranged from 0.75–0.90 between assessor 1 and 2, 0.68–0.96 between assessor 1 and the algorithm, and 0.75–0.91 between assessor 2 and the algorithm (*p* < 0.01 for all). The lowest ICC was shown for the diaphragm/intercostal and the highest for the parasternal intercostal (0.68 and 0.96, respectively). During ITL, diaphragm/intercostal EMG, onset occurred later during the inspiratory cycle and its activity duration was shorter than the scalene, parasternal intercostal, and sternocleidomastoid (*p* < 0.01). EMG, offset occurred synchronously across all muscles (*p* ≥ 0.98). EMG, onset, and EMG, offset timing, and activity duration was consistent throughout the ITL for all muscles (*p* > 0.63).

**Conclusion:** We developed an algorithm to detect EMG, onset of several respiratory muscles with millisecond accuracy that is time-efficient and validated against manual measures. Compared to the inherent bias of manual measures, the algorithm enhances objectivity and provides a strong standard for determining the respiratory muscle EMG, onset.

## Introduction

Respiratory muscle activity to generate ventilation is mainly automated under the control of the respiratory centers located in the pontomedullary region of the brainstem (Feldman and Del Negro, 2006; Hudson et al., 2016). The respiratory centers’ output to the respiratory muscle will determine whether a muscle is active, regulates the amplitude of its activation, and coordinates the timing of its activity. In humans, the respiratory centers’ output cannot be directly measured (Vaporidi et al., 2020). The respiratory drive to the respiratory muscles measured *via* electromyography (EMG) is used as its surrogate (Domnik et al., 2020). Respiratory muscle EMG allows the identification of whether a muscle is active and provides a relative indication of the amplitude of the muscle electrical activity [e.g., the root mean square (RMS) of the EMG signal]. In addition, the timing of activation can be evaluated by the onset and offset of their activity (EMG, onset and EMG, offset, respectively; Epiu et al., 1985; Nguyen et al., 1985; Hodges and Gandevia, 2000; Luo and Moxham, 2005; Hudson et al., 2011, 2016; Sinderby et al., 2013; Estrada et al., 2019; Domnik et al., 2020; Vaporidi et al., 2020).

Measurements of the respiratory drive *via* respiratory muscle EMG have now been used for over 100 years (Domnik et al., 2020). Most studies have focused on the amplitude of the respiratory muscle activation (e.g., EMG RMS), while the coordination between the timing of different respiratory muscle activity (e.g., their EMG, onset, EMG, offset, and duration of activity) has been less investigated. Most studies that have investigated the coordination between different respiratory muscle EMG, onset, EMG, offset, and duration of activity during the respiratory cycle have visually identified when the inspiratory modulation of the EMG activity began and ended (Epiu et al., 1985; Nguyen et al., 1985; Hodges and Gandevia, 2000; Hudson et al., 2011, 2016; Sinderby et al., 2013). This approach also allows to quantify the duration of the muscle activity in relation to the respiratory cycle (Epiu et al., 1985; Nguyen et al., 1985; Hodges and Gandevia, 2000; Hudson et al., 2011, 2016; Sinderby et al., 2013). However, this method can be time-consuming, especially for recordings that contain many breathing cycles (e.g., during exercise). Also, this method is prone to subjective interpretations to determine “when the modulation of the EMG activity began.” It could lead to between-assessors variability in defining when the modulation of the EMG signal occurred.

We have previously shown that such subjective interpretation can influence effect size estimates in pre- vs. post- interventional studies of EMG data (Dacha et al., 2019). Moreover, the respiratory muscle EMG is highly contaminated by electrocardiogram (ECG) artifacts (Luo et al., 2008; Dacha et al., 2019; Domnik et al., 2020). In breaths where the EMG, onset coincides with the ECG artifact, the detection of the EMG, onset is not possible. Filters that exclude the part of the EMG signal that is contaminated by the ECG artifacts have been applied (Sinderby et al., 1985; Luo et al., 2008; Hudson et al., 2016). However, these could lead to a time difference of 0.10–0.12 s in the EMG, onset detection – based on the length of the QRS complex of the ECG signal. For example, this could be equivalent to approximately 10% of the inspiratory time at a rate of ≈20 breaths/min and 1 s inspiratory time, and an even greater proportion of the cycle with faster respiratory rates (e.g., during exercise). Despite being small, these differences could either under- or overestimate breath-by-breath or between muscle comparison of the EMG, onset, EMG, offset, or activity duration. For instance, the 5th dorsal external intercostal has been described as the latest intercostal activated during inspiration, at approximately 14.5% of the inspiratory time, compared to the costal diaphragm at −2.5% or the 3rd external intercostal at −1.0% in measurements performed using needle EMG (Saboisky et al., 1985; Hudson et al., 2011).

We previously developed an algorithm that filters out ECG artifacts from the EMG signal of the respiratory muscle without the need to exclude any segment of the EMG signal (Dacha et al., 2019). Herein, our main objective, building on the previous version of this algorithm, was to develop and validate a method to investigate the EMG, onset of different respiratory muscles in an objective and time-efficient manner. Specifically, we aimed to: (1) develop a semi-automated algorithm to detect the EMG, onset of the respiratory muscles; (2) determine the interrater reliability of the manual method of detecting the EMG, onset of the respiratory muscles; (3) validate the EMG, onset detected by our semi-automated algorithm against manual measures. Secondly, we applied the algorithm to explore (1) EMG, onset differences among four respiratory muscles during a constant load inspiratory threshold loading (ITL) task performed to task failure in healthy young adults, and (2) whether the timing of the EMG, onset is affected by the intensity of the inspiratory effort, the peak inspiratory flow or the amplitude of the EMG activity.

## Materials and Methods

Healthy adults aged between 18 and 35 years were included in this study. All participants provided written informed consent at the time of enrollment. Participants were enrolled between March and July 2017. The conduct of the analysis presented herein was approved by the University of Toronto Health Sciences Research Ethics Board (#39041). EMG and ventilatory parameters were recorded during an ITL performed to task failure. During the ITL, participants were cued to target a respiratory rate of 10 breaths per minute by listening to an audio recording but were not instructed to begin inspirations from a particular lung volume. The participants performed the ITL until task failure at 50% of their maximum inspiratory pressure (MIP) by inhaling against a spring-loaded threshold device [PowerBreathe^™^, Classic MR (range 10–90 cmH_2_O), International Ltd., England, United Kingdom] connected to a two-way non-rebreathing valve (Hans Rudolph, Kansas City, MO) in line with a mouthpiece with a port connected to a differential pressure transducer (MP45-36-871; Validyne^™^, Northridge, CA). Task failure was defined as when the participants took their mouth off the mouthpiece or could no longer overcome the spring-loaded threshold valve for inspiration for three consecutive breaths.

Patients’ age (years), height (cm), and mass (kg) were determined. Body mass index (BMI; kg m^−2^) was calculated. The forced volume in the first second (FEV_1_; ml) and forced vital capacity (FVC; ml) were measured by spirometry according to international guidelines (Miller et al., 2005) and the FEV_1_/FVC ratio was calculated. Dyspnea was measured before the ITL and at task failure using the modified Borg score (Borg, 1982). MIP was measured before the ITL according to the standardized procedure (American Thoracic Society/European respiratory Society, 2002; Laveneziana et al., 2019) except that participants were in a half-lying position. Participants were instructed to forcefully inspire after having performed a full expiration to residual volume while breathing through a flanged mouthpiece connected to an occluded three-way stopcock (2100 series, Hans Rudolph, Kansas City, MO) with a small port connected to a pressure transducer (MP45-36-871; Validyne^™^, Northridge, CA). MIP was measured 3 to 10 times until the variability between the three highest values was ≤10%. MIP was expressed in absolute values (cmH_2_O) and as percent of the predicted (%) according to Evans and Whitelaw (2009).

Surface electromyography (EMG) was acquired throughout the ITL to measure muscle activation from the costal diaphragm/intercostals, scalene, parasternal intercostal, and sternocleidomastoid. The participant’s skin was prepared with shaving when necessary and cleaned with alcohol. EMG signals were acquired by placing electrodes 2.5 cm apart on the right hemithorax overlying: (1) the costal diaphragm/intercostals at the seven or eight intercostal space between the anterior axillary line and midclavicular line in accordance with the best signal captured; (2) the scalene in the posterior triangle of the neck at the level of the cricoid process; (3) the parasternal intercostals on the second intercostal space close to the sternum; (4) the sternocleidomastoid midway between the suprasternal notch and the mastoid process. EMG was acquired using an eight-channel bioamplifier (BioAmp; ADInstruments, Colorado Springs, CO), converted to digital signals (PowerLab; ADInstruments), and recorded at 1000 Hz by a data acquisition software (LabChart, ADInstruments, Colorado Spring, CO).

Ventilatory parameters, including mouth pressure (Pm), respiratory frequency (*fr*), inspiratory flow, and inspiratory tidal volume (*vt*), acquired by a pneumotach connected between the threshold loading device and the inspiratory port of the two-way non-rebreathing valve of the ITL apparatus, and electrocardiography signals (ECG), were acquired synchronously with the EMG throughout the ITL using the same system (PowerLab; ADInstruments) and recorded by the same data acquisition software (LabChart, ADInstruments, Colorado Spring, CO).

### The Algorithm to Detect the EMG Onset

EMG, inspiratory flow, and ECG signals collected throughout the ITL were exported from the data acquisition software (LabChart, ADInstruments, Colorado Spring, CO) at 1000 Hz into a text file. This file was imported into the LABVIEW software (National Instruments, Austin, TX, United States) in which an in-house algorithm was developed by a biomedical engineer (LJ) to first filter out ECG artifacts from the EMG signals and then automatically detect the onset of each muscle EMG activity breath-by-breath. The development and validation of the ECG removal process have been previously published by our group (Dacha et al., 2019). Briefly, we used a bidirectional high pass filter at 20 Hz 2nd order Butterworth. A bidirectional filter was applied to avoid lagging and/or leading the EMG signals as it would have occurred if a unidirectional filter had been used, especially at low frequencies, potentially affecting the timing of the EMG, onset (Willigenburg et al., 2012). The ECG artifacts were removed from the EMG signals using the least mean square adaptive filter (Dacha et al., 2019). This is a pattern recognition method that uses a time-aligned ECG signal to remove the ECG frequency content from the EMG signals without the need of deleting any part of the EMG signal, which would also potentially alter the timing of the EMG, onset.

The ECG filtered EMG signals from each muscle were transformed into root mean square (RMS) and the first derivative function of each muscle EMG RMS was calculated. Based on the derivative function of each muscle EMG RMS, we identified the “rising” and “descending” phases of the EMG RMS. While a positive derivative indicates a rising EMG RMS, a negative derivative indicates a descending EMG RMS. It was necessary to use the derivative function to determine the rising and descending phases of the EMG RMS because: (1) the “baseline” EMG RMS varied from participant to participant and (2) the EMG RMS does not always return to the “baseline” after an activity burst. Therefore, identifying the rising and descending phases of the EMG RMS based on an absolute value would not consistently detect timing of the EMG onset and offset.

**Figure 1:**
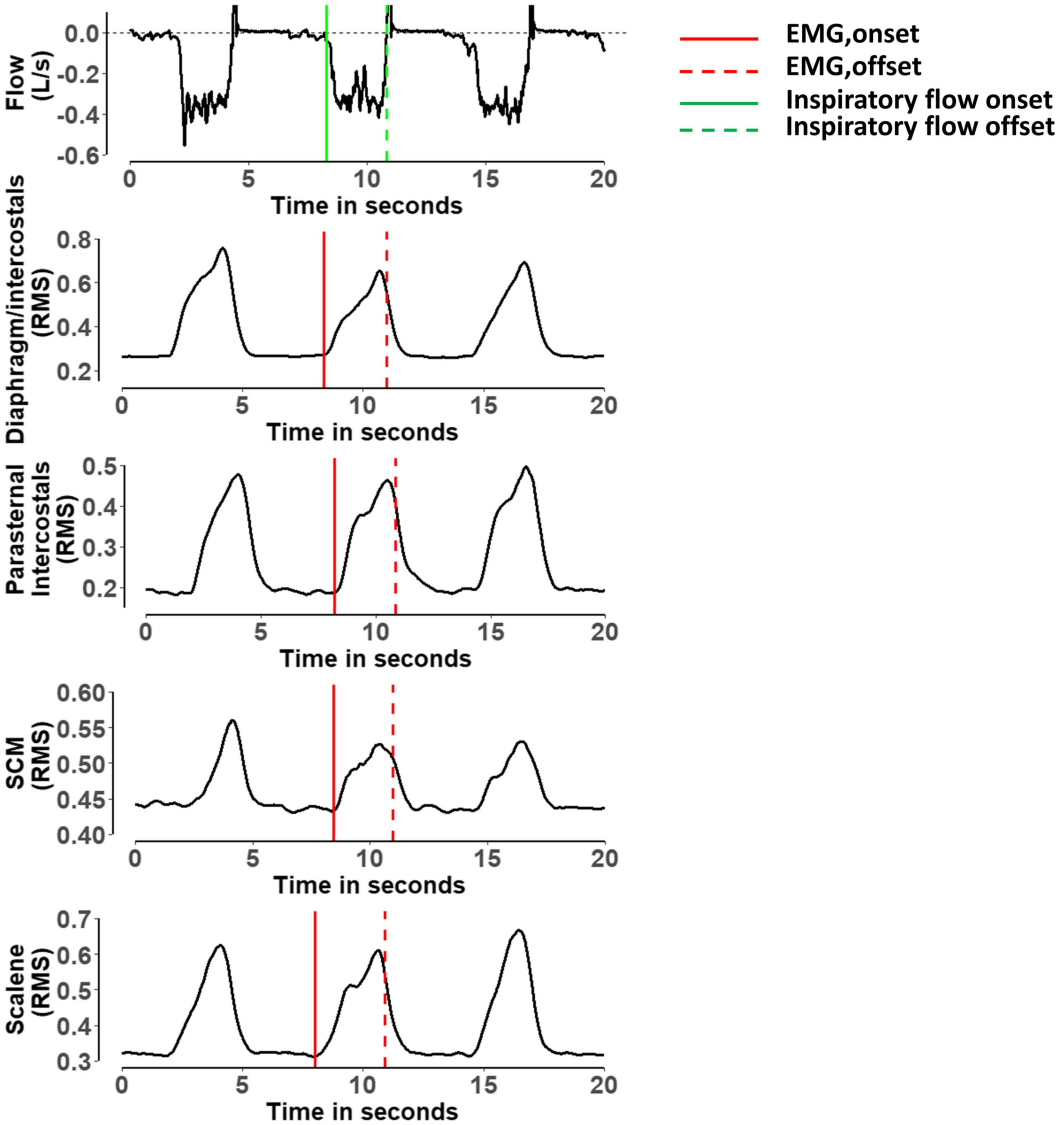
Representative breath showing the detection of the onset and offset of the electromyography activity for the diaphragm/intercostal, parasternal intercostal, sternocleidomastoid and scalene. RMS, root mean square of the electromyography; SCM, sternocleidomastoid. Red lines indicate when the algorithm detected the onset of the electromyography activity for each muscle. Dashed red lines indicate when the algorithm detected the offset of the electromyography activity for each muscle. Green line indicates when the algorithm detected the onset of the inspiratory flow. Dashed green line indicate when the algorithm detected the offset of the inspiratory flow (Sinderby et al., 2013; Estrada et al., 2019).

Using the flow signal, we identified the beginning of each breath’s inspiratory phase (INSP, onset) within ±1 msec accuracy. The maximum value of the rise of the EMG RMS was identified breath-by-breath. The onset time of the EMG activity (EMG, onset) was defined as the timepoint when the rise of the EMG RMS reached 5% of its maximum (±1 msec). We used a 5% threshold to detect the EMG, onset to avoid the inherent variability of the baseline EMG signal to be mistakenly identified as activation. The EMG, onset could occur either before or after the beginning of the INSP, onset depending on when the 5% threshold occurred. The EMG filtering and the EMG, onset detection could be carried out in parallel for EMG signals from multiple muscles. Figure 1 shows the EMG, onset detection for the diaphragm/intercostal, scalene, parasternal intercostal, and sternocleidomastoid in a representative breath. One additional function was implemented to the algorithm to detect the offset of the neural inspiratory drive to the respiratory muscles (EMG, offset) using thresholds previously validated in the literature (Sinderby et al., 2013; Estrada et al., 2019). EMG, offset was defined as when the EMG RMS dropped by 30% after reaching its peak (Sinderby et al., 2013; Estrada et al., 2019). The analyzed data were then exported from the LABVIEW software (National Instruments, Austin, TX, United States) to a text file containing each muscle EMG, onset and EMG, offset and INSP, onset and INSP, offset for each breath. The text file was saved for statistical analysis.

### Validation of the Algorithm to Detect the EMG Onset

**Figure 2:**
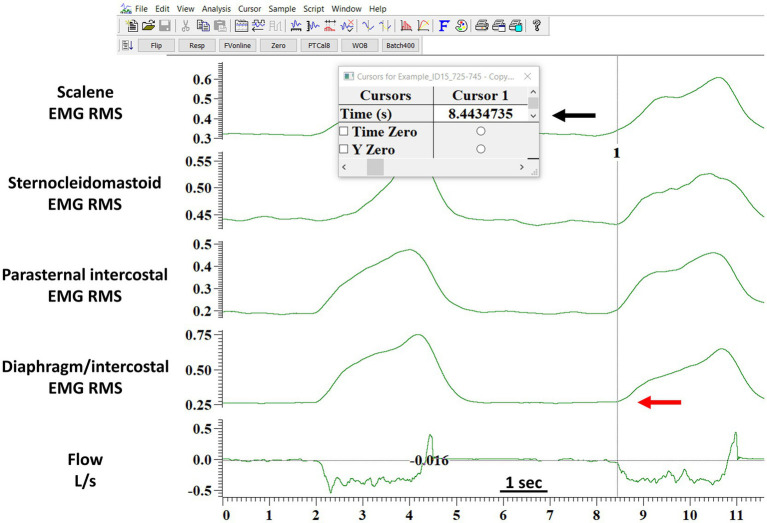
Example of the detection of the onset of the EMG activity by the manual method. The figure shows the software layout viewed by the assessors when performing the manual analysis. The red arrow indicates where the assessor judged the onset of the diaphragm/intercostals EMG onset occurred. The black arrow indicates the time in seconds (provided by the software) when the assessor indicated the diaphragm/intercostal EMG onset occurred. The assessor would type the seconds indicated by the software in a Excel file with a millisecond accuracy. This processed was repeated breath-by-breath for each muscle. EMG RMS, root mean square of the EMG signal; DIA/IC, diaphragm/intercostal; PI, parasternal intercostal; SCM, sternocleidomastoid.

**Figure 3:**
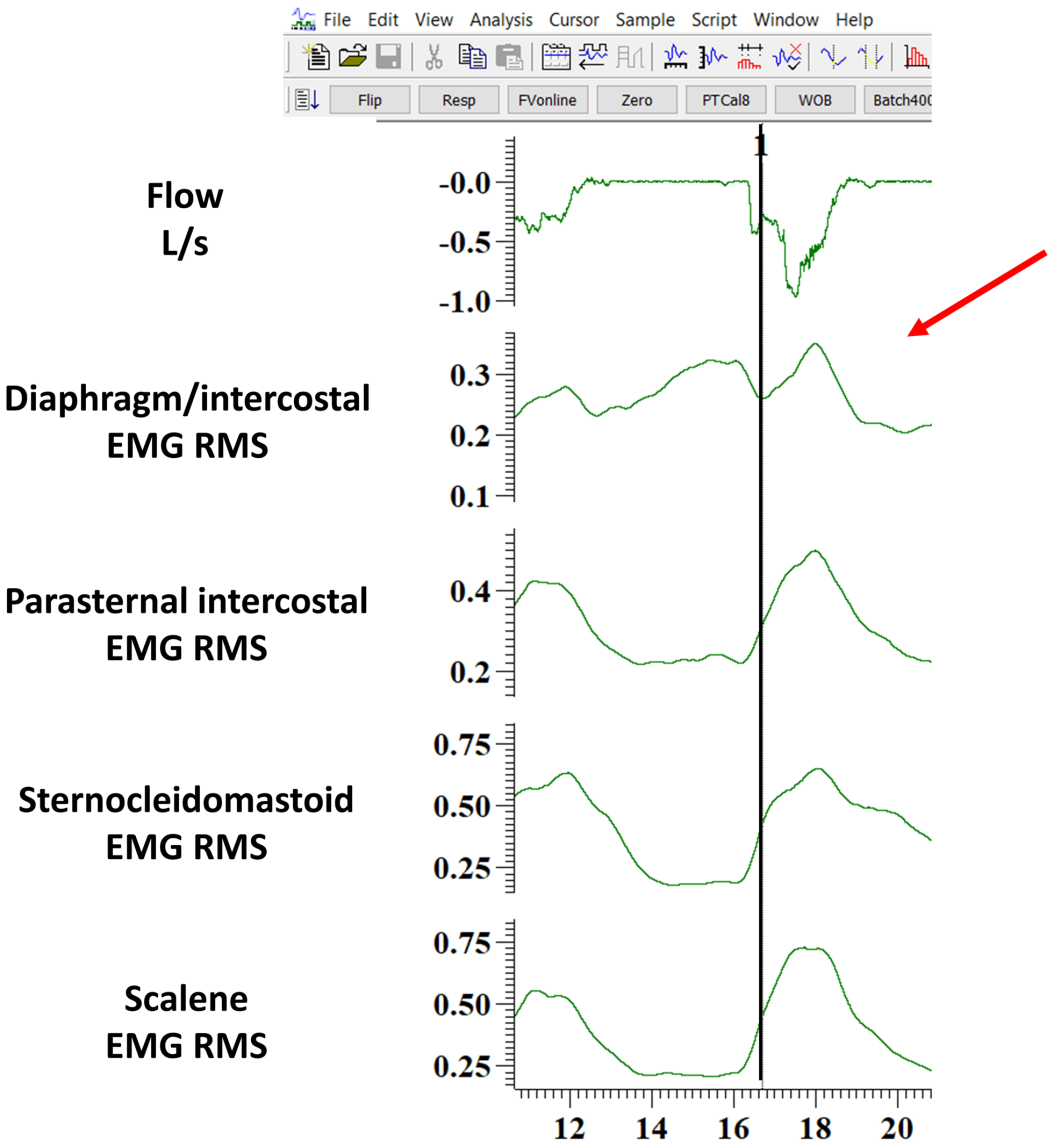
Example of one breath where the diaphragm/intercostal EMG onset was judged not clear by the assessor (indicated by the red arrow) and thus typed as “NA” in the Excel file. The figure shows the software layout viewed by the assessor when performing the manual analysis. The black line indicates where the algorithm identified the beginning of the EMG onset. EMG RMS: root mean square of the EMG signal; DIA/IC, diaphragm/intercostal; PI, parasternal intercostal; SCM, sternocleidomastoid.

The validation of the algorithm was performed by random sampling 10 min of data from each participant’s ITL. EMG signals from all muscles, ECG, and flow signals during the ITL were available. First, we used the algorithm to detect the EMG, onset for each muscle breath-by-breath as described above. Second, the EMG, onset was detected manually by two independent assessors. Both assessors were provided with the ECG filtered EMG RMS to perform the manual analysis (see above). Both assessors opened the ITL files using a dedicated software (Spike 2, Cambridge Electronic Design Limited, Cambridge, United Kingdom). For each breath, both assessors were instructed to determine each muscle’s EMG, onset, as defined by a visual increase in the EMG RMS, by placing a cursor at the location where they judged the onset occurred. The software then provided the assessors with the timing at which they placed the cursor. Figure 2 shows an example of the process for detecting one EMG onset. For each breath, both assessors typed each muscle EMG, onset time with a millisecond’s accuracy into a Excel file that was saved for posterior analysis. Hence, we could posteriorly calculate the interrater reliability between the manual method performed by two independent assessors, and the validity was studied by determining the agreement between each assessor and the algorithm (see below for more details). Both assessors were blinded to each other results. Of note, if the EMG, onset of any muscle could not be identified for a given breath due to artifacts, small amplitude or any other reason, both assessors were instructed to type “NA” instead of the EMG, onset time on the Excel file. Figure 3 shows an example of a breath that the EMG, onset was judged not clear for the diaphragm/intercostal and thus not identified using the manual method. Additionally, both assessors recorded the time required for their manual analyses. Both assessors had at least 1-year experience analyzing EMG signals, have received previous training and practiced detecting the onset of the EMG signal for each of these four muscles in at least 300 breaths (total of at least 1200 onset detections) before performing the analysis reported herein. We did not perform the validation of the EMG, offset because the threshold we implemented in our algorithm has already been validated in physiological studies (Sinderby et al., 2013; Estrada et al., 2019) and is widely used in the clinical setting (e.g., to detected the offset of the neural drive in mechanically ventilated patients using neurally adjusted ventilatory assist).

### Timing of Respiratory Muscles EMG Onset Relative to Flow

For each breath and each respiratory muscle, the absolute and relative timing differences between EMG RMS and inspiratory flow or were determined as follows: (1) EMG, onset and the INSP, onset and (2) EMG, offset, and INSP, offset. Absolute differences were calculated for the phase difference (*dP*), in milliseconds whereas relative time differences were determined by normalizing the *dP* to the duration of the inspiratory time based on the flow signal (%Ti), as previously described (Nguyen et al., 1985; Hodges and Gandevia, 2000; Hudson et al., 2011, 2016). The normalization was performed to account for breath-to-breath variations in the inspiratory time and to allow us to compare our results to those previously published in the literature (Nguyen et al., 1985; Hodges and Gandevia, 2000; Hudson et al., 2011, 2016).

The following equations were applied to each breath for each muscle’s EMG RMS:

1. Absolute difference between EMG onset and inspiratory flow (or Pm):[Respiratorymuscle],dPon(ms)=EMG,onset(ms)−INSP,onset(ms)
2. Absolute difference between EMG offset and the end of inspiratory flow (or Pm):[Respiratorymuscle],dPoff(ms)=EMG,offset−INSP,offset(ms)
3. Relative difference between EMG onset and inspiratory flow (or Pm):[Respiratorymuscle],%Tion=([Respiratorymuscle],dPon÷Ti)×100
4. Relative difference between EMG offset and the end of inspiratory flow (or Pm):[Respiratorymuscle],%Tioff=([Respiratorymuscle],dPoff÷Ti)×100

For the timing differences in EMG, onset or EMG, offset in milliseconds (*dP*) or normalized by the inspiratory time (%Ti), a value of zero indicates synchrony of the EMG and flow signal or mouth pressure, that is, EMG, onset, and INSP, onset or EMG, offset and INSP, and offset; if less than zero, it indicates that the change in the EMG signal preceded the flow signal or pressure signal; and if greater than zero, it indicates that the change in the EMG signal occurred after the flow signal or pressure signal.

### Timing of Respiratory Muscles EMG Onset and Offset During the ITL

After the algorithm was developed and validated, we analyzed the ITL trials from start up to isotime and during task failure to explore EMG, onset, and EMG, offset differences among respiratory muscles during ITL. Isotime was defined as the highest equivalent time achieved by all participants during the ITL rounded to the nearest minute. Task failure was defined as the last 2 min of each participant’s ITL. In addition to the EMG, onset, and EMG, offset, we also analyzed Pm as surrogate for inspiratory muscle effort. Additionally, because the ITL can introduce variability in the delay between the EMG, onset, and INSP, onset we also analyze the timing of EMG activity based on the pressure signal. INSP, onset, peak *Pm*, peak inspiratory flow, *vt* and diaphragm/intercostals, scalene, parasternal intercostal, and sternocleidomastoid EMG, onset, and EMG, offset were determined breath-by-breath during the ITL using the algorithm. For each breath, diaphragm/intercostals, scalene, parasternal intercostal and sternocleidomastoid EMG, onset, and EMG, offset in milliseconds and normalized by the duration of the inspiratory time (based on both flow and pressure signals) were calculated as described in equations 1–4. We also calculated the duration of activation during each muscle contraction breath-by-breath, defined as the time elapsed between each muscle EMG, onset and EMG, offset in both milliseconds and normalized by the duration of the inspiratory time (based on both flow and pressure signals). Diaphragm/intercostals, scalene, parasternal intercostal and sternocleidomastoid EMG, onset, EMG, offset, the duration of their activation, Ttot, inspiratory time, peak inspiratory flow, peak *Pm*, and *vt* were averaged every 2 min during the ITL up to isotime and at task failure to evaluate the time course of these variables.

### Statistical Analysis

No statistical power or sample size calculations were conducted *a priori* since this was a secondary analysis (Derbakova et al., 2020). Ten minutes of ITL data from each participant for the validation of the algorithm was selected because it provided approximately 100 breaths. Descriptive statistics were reported as frequencies, mean ± SD or median (25–75% IQR) according to the data distribution assessed using the Shapiro–Wilk test unless otherwise stated. For the validation of the algorithm, the interrater reliability between assessor 1 and 2 and between each assessor with the algorithm was calculated using the two-way mixed effects Intraclass Correlation Coefficient (ICC) and visualized using Bland-Altman plots. ICCs and Bland and Altman plots were done for each muscle independently in milliseconds and normalized by the duration of the inspiratory time. Interrater reliability was classified as “poor” (ICC < 0.5), “moderate” (ICC 0.5–0.75), “good” (ICC 0.75–0.9), or “excellent” (ICC > 0.9).

To assess differences of the timing of the respiratory muscle EMG, onset and EMG, offset during the ITL, two-way ANOVAs were conducted to test the main effect of “time” (ITL start up vs. isotime vs. task failure as factor 1) and “between muscle” (factor 2) and their interaction. Similar two-way ANOVAs were also performed on EMG RMS and the duration of activation. Repeated measures ANOVAs were used to test the main effect of time (from ITL start up to isotime and during task failure) on the Ttot, inspiratory time, inspiratory flow, *vt* and *Pm*. Repeated measures ANOVAs were also used to compare the EMG, onset of each muscle between the ITL start, isotime, and task failure. *Post hoc* testing for significant variables was carried out using Tukey adjustment for multiple comparisons. Paired *t* test was used to compare Borg scores before the ITL and at task failure. The chi-square test was used for comparing frequencies. Density plots using kernel density estimates were built to show the distribution of the diaphragm/intercostals, scalene, parasternal intercostal and sternocleidomastoid EMG, onset normalized by the duration of the inspiratory time at the start, isotime, and peak ITL. Boxplots were built to supplement the density plots visualization. Pearson correlation coefficient (*r*) was used to assess whether the timing of each muscle EMG, onset, or the duration of their activation was associated with the magnitude of the inspiratory effort (*Pm*), the peak inspiratory flow, or the amplitude of their EMG activity (EMG RMS). The strength of the correlations (*r*) were classified as “small” (0.1–0.3), “medium” (0.3–0.5), or “large” (0.5–1). A *p* < 0.05 was considered statistically significant for all analyses.

## Results

### Participants’ Characteristics

Seven participants were included. Participant’s characteristics are presented in Table 1. Participants’ age was 24 ± 1 years, their body composition was normal according to their BMI, and they had preserved FEV_1_/FVC ratio. Four were male. MIP was 90 ± 13% of predicted values (Evans and Whitelaw, 2009).

**Table 1:** Participant characteristics (*n* = 7).

Age, years	24 ± 1
Sex, male/female	4/3
BMI, kg m^−2^	24 ± 3
MIP, cmH_2_O	99 ± 16
MIP, %pred (Evans and Whitelaw, 2009)	90 ± 13
FVC, L	4.2 ± 0.67
FEV_1_, L	3.4 ± 0.58
FEV_1_/FVC	0.82 ± 0.07

*BMI, body mass index; MIP, maximal inspiratory pressure; FVC, forced vital capacity; FEV_1_, Forced expiratory volume in the first second*.

### Validation of the Algorithm

**Figure 4:**
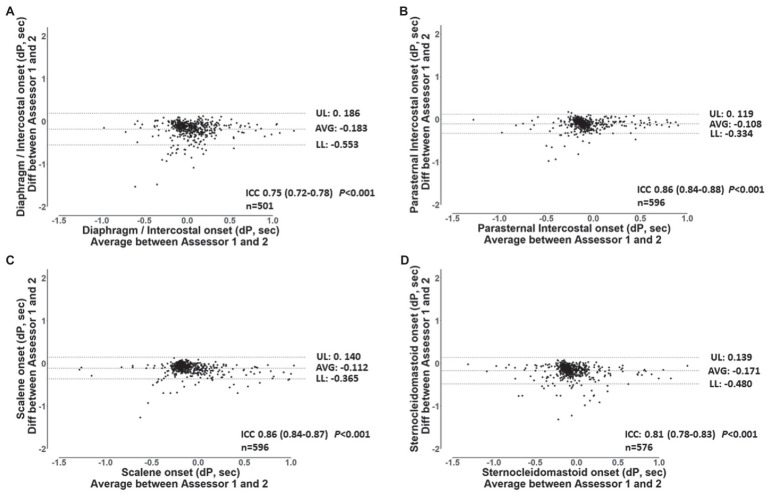
Bland-Altman plots and ICC’s values for the breath-by-breath detection of *dP* of EMG, onset by assessor 1 and 2 for the diaphragm/intercostal **(A)**, parasternal intercostal **(B)**, scalene **(C)** and, sternocleidomastoid **(D)**. AVG: average bias between the results from both assessors. UL and LL: 95% confidence interval of the difference between the results from both assessors. n: number of EMG, onset analyzed. *dP*: phased difference between the onset of the electrical activity of the muscle and the start of the inspiration calculated (see text for further details).

**Figure 5:**
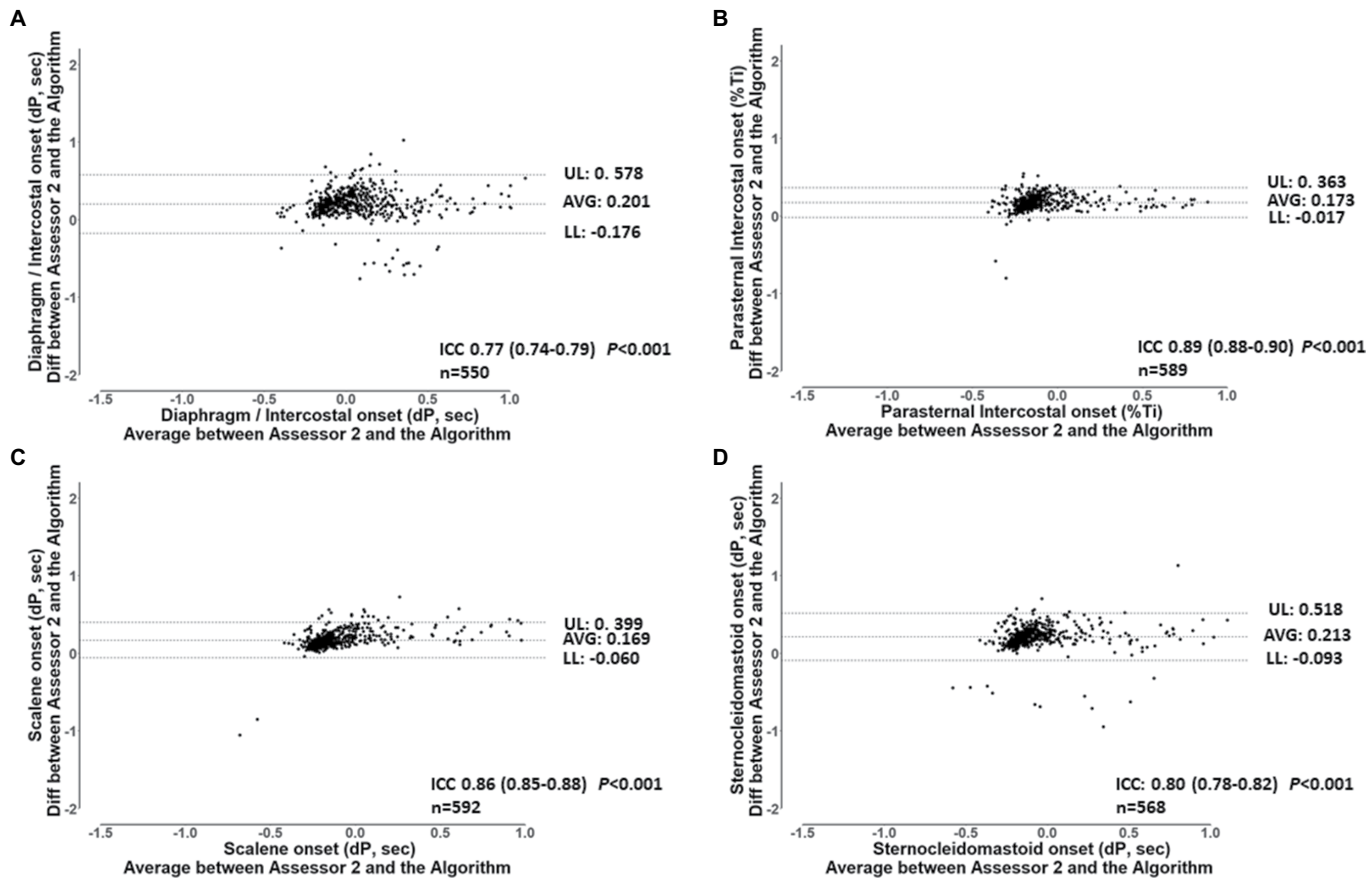
Bland and Altman plots and ICC’s values for the breath-by-breath detection of *dP* of EMG, onset by assessor 1, and the algorithm for the diaphragm/intercostal **(A)**, parasternal intercostal **(B)**, scalene **(C),** and sternocleidomastoid **(D)**. AVG: average bias between the results from both assessors. UL and LL: 95% confidence interval of the difference between the results from both assessors. n: number of EMG, onset analyzed. *dP*: phased difference between the onset of the electrical activity of the muscle and the start of the inspiration calculated (see text for further details).

For the validation of the semi-automated algorithm, 70 min of data from the 7 participants were analyzed. Ten minutes of data randomly selected from each participant’s ITL generated 92 ± 4 breaths per participant resulting in a total of 646 breaths and 2576 EMG RMS signals (7 participants × 92 breaths × 4 muscles). Table 2 shows the number of EMG, onset detected by the algorithm and by each assessor for each muscle. A total of 2,486 EMG, onset were detected by the algorithm, 2376 by assessor 1 and 2403 by assessor 2. Assessor 1 detected significantly fewer EMG, onset for the diaphragm/intercostal than both the algorithm and assessor 2 (*p* < 0.05). Assessor 2 detected less EMG, onset for the scalene, parasternal intercostal, and sternocleidomastoid than both assessor 1 and the algorithm (*p* < 0.05). Figure 4 shows a representative Bland-Altman plot with the interrater reliability between Assessor 1 and 2 and Figure 5 between Assessor 1 and the algorithm, while Bland-Altman plots for each muscle between Assessor 1 and 2 and each assessor and the algorithm are shown in the online supplement (Supplementary Figures S1–S6). Overall, interrater reliability for detecting the EMG, onset was classified as good between Assessor 1 and 2 (Figure 4; Supplementary Figures S1, S2), moderate to excellent between assessor 1 and the algorithm (Figure 5; Supplementary Figures S3, S4), and good to excellent between assessor 2 and the algorithm (Supplementary Figures S5, S6; *p* < 0.001 for all). Mean times required per analysis were greatest for Assessor 2, intermediate for Assessor 1, and least for the algorithm (174 ± 10 min vs. 66 ± 13 min vs. 1 ± 0 min, respectively; *p* < 0.001 between all).

**Table 2:** Number of EMG, onset detected for each muscle by the algorithm and Assessor 1 and 2 from all 646 breaths used for validating the algorithm.

	Algorithm	Assessor 1	Assessor 2
EAdi, onset (n)	624	506^*^	569^*^^,^^¥^
EAsca, onset (n)	627	631	615^*^^,^^¥^
EApi, onset (n)	624	626	613^¥^
EAscm, onset (n)	611	613	606^¥^

* *p < 0.05 vs. the Algorithm*.

¥ *p < 0.05 vs. Assessor 1*.

### Timing of Respiratory Muscles EMG Onset and Offset During the ITL

**Figure 6:**
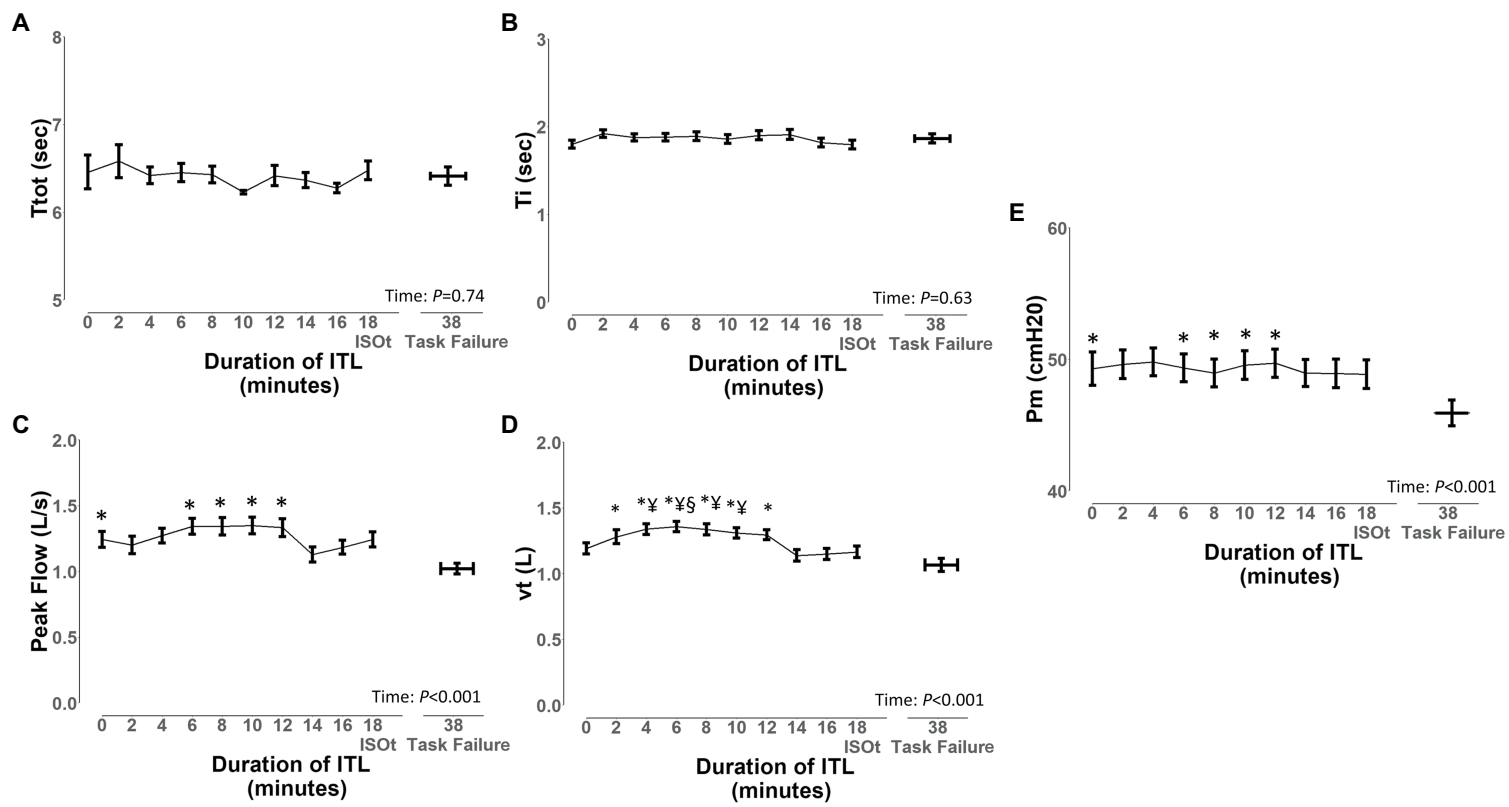
Changes in ventilatory variables during the inspiratory threshold loading. Ttot, total time of the respiratory cycle **(A)**, Ti, inspiratory time **(B)**, peak flow, peak inspiratory flow **(C)**, vt, tidal volume **(D),** and *Pm*, mouth pressure **(E)**. Data are depicted as mean ± SE. ^*^p < 0.05 vs. task failure; ^§^*p* < 0.05 vs. minutes 14 and 16; ^¥^*p* < 0.05 vs. minute 18.

To assess the diaphragm/intercostal, scalene, parasternal intercostal, and sternocleidomastoid EMG, onset and EMG, offset during the ITL, we analyzed the ITL data from start up to isotime and during task failure from the 7 participants. A total of 1,434 breaths were analyzed (401 ± 144 breaths per participant). ITL load was 49 ± 8 cmH_2_O, isotime was 18 min, and mean time to task failure was 38 ± 13 min. Borg scores increased from baseline to task failure (0 ± 0 vs. 4 ± 2; *p* = 0.001). Ttot and inspiratory time (Figure 6) did not significantly change from ITL start to task failure (*p* = 0.74 and *p* = 0.63, respectively). Peak inspiratory flow was reduced at task failure compared to ITL start and minutes 8, 10 12, and 14 (Figure 6; *p* < 0.05), *vt* was reduced at minute 14 and 16 compared to minute 8 and at task failure compared to minutes 2, 4, 6, 8, 10 and 12 (Figure 6; *p* < 0.05). Diaphragm/intercostals, scalene, parasternal intercostal, and sternocleidomastoid EMG RMS (Supplementary Figure S7) did not significantly change from ITL start to task failure (*p* = 0.96). EMG RMS was lower in the diaphragm/intercostal than in the scalene, parasternal intercostal, and sternocleidomastoid throughout the ITL (Supplementary Figure S7; *p* < 0.001). EMG RMS was also lower in the parasternal intercostal than in the scalene (Supplementary Figure S7; *p* = 0.02).

**Figure 7:**
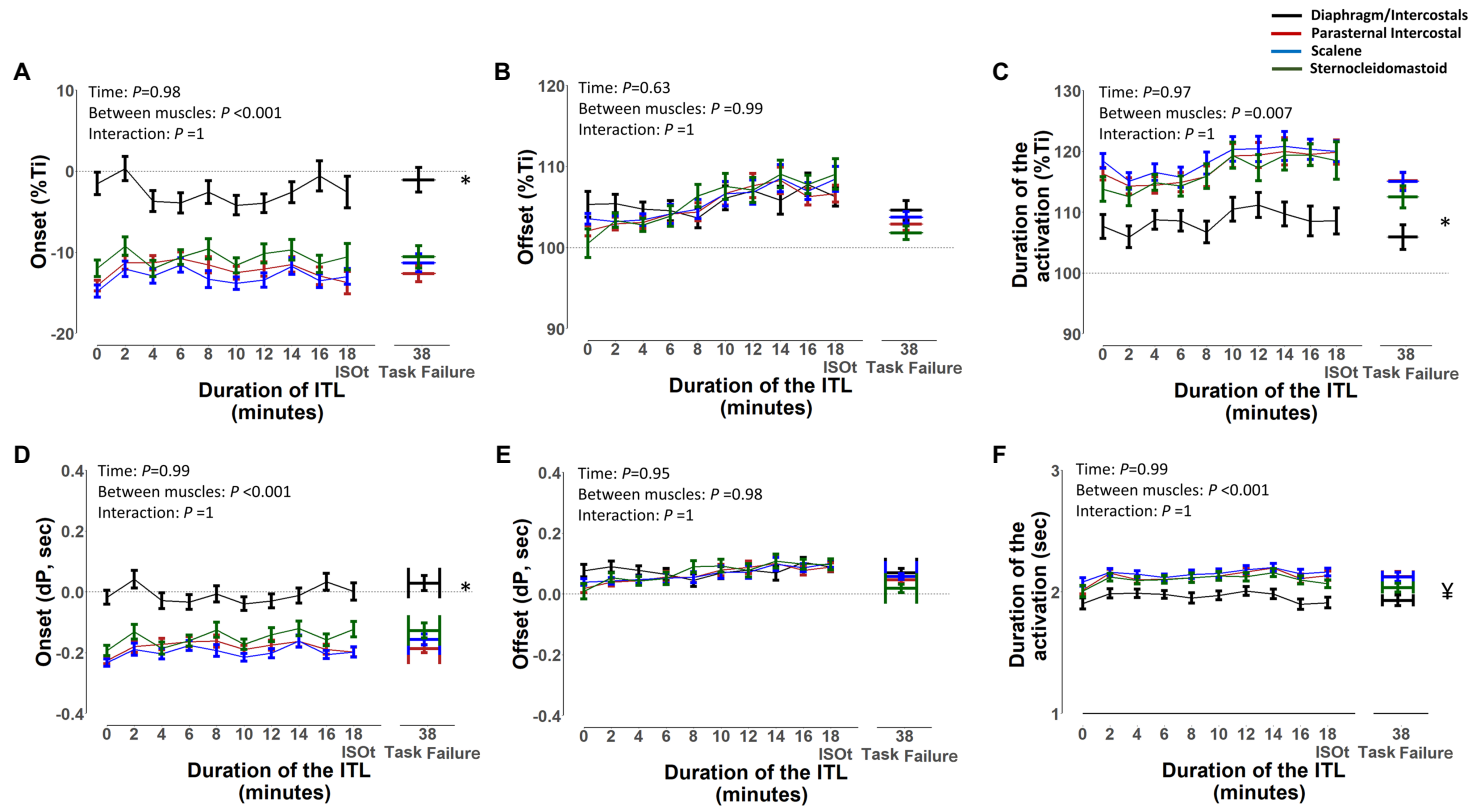
Timing of the diaphragm/intercostal, parasternal intercostal, scalene, and sternocleidomastoid activity during the inspiratory threshold loading. Upper panels are expressed as a percentage of inspiratory time onset based on the flow signal (%Ti) whereas lower panels are expressed in seconds from inspiratory flow onset **(A)** and **(D)** – EMG, onset; **(B)** and **(E)** – EMG, offset; **(C)** and **(F)** – Duration of activation. ^*^p < 0.01 for the diaphragm/intercostal vs. parasternal intercostal, scalene and sternocleidomastoid. ^¥^*p* < 0.03 for the diaphragm/intercostals vs. parasternal intercostal and scalene.

**Figure 8:**
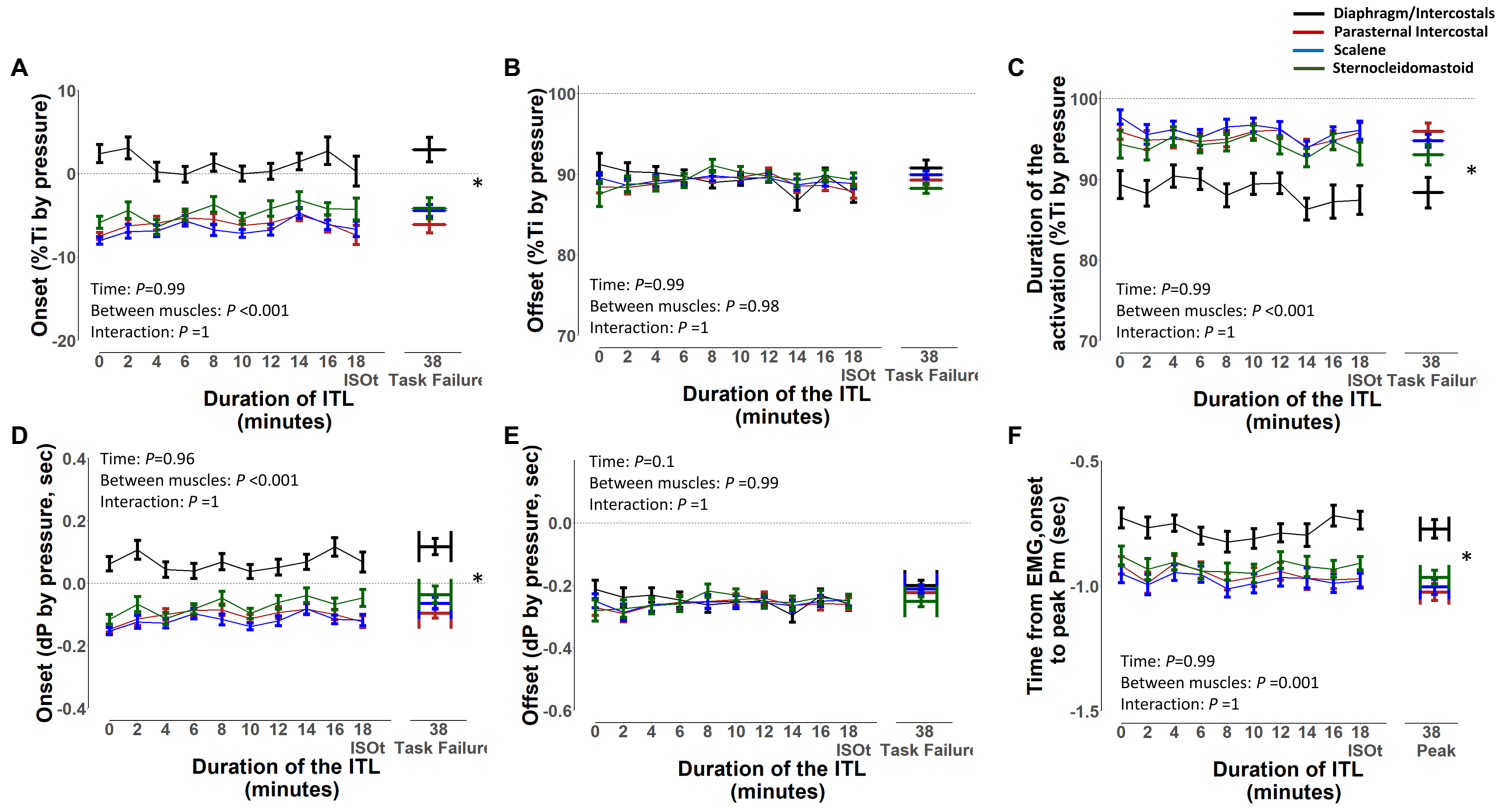
Timing of the diaphragm/intercostal, parasternal intercostal, scalene, and sternocleidomastoid activity during the inspiratory threshold loading. Upper panels are expressed as a percentage of inspiratory time onset based on the pressure signal (%Ti by pressure) whereas lower panels are expressed in seconds from inspiratory onset based on the pressure signal **(A)** and **(D)** – EMG, onset; **(B)** and **(E)** – EMG, offset; **(C)** and **(F)** – Duration of activation. ^*^p < 0.01 for the diaphragm/intercostal vs. parasternal intercostal, scalene and sternocleidomastoid.

The EMG, onset, and EMG, offset and duration of activation of the diaphragm/intercostal, scalene, parasternal intercostal, and sternocleidomastoid in milliseconds and normalized by the duration of the inspiratory time based on both flow and pressure signals are shown in Figures 7, 8, respectively. The EMG, onset, and EMG, offset of all muscles did not change throughout the ITL (*p* ≥ 0.63). However, the EMG, onset of the diaphragm/intercostal was significantly greater (later) than the scalene, parasternal intercostal, and sternocleidomastoid (*p* < 0.001; Figures 7, 8). The EMG, offset occurred synchronously across all muscles (*p* ≥ 0.98). The duration of the activation did not change throughout the ITL for all muscles (*p* ≥ 0.97). However, the diaphragm/intercostal duration of activation normalized by the duration of the inspiratory time based on both flow and pressure signal was shorter than the parasternal intercostal, scalene, and sternocleidomastoid (*p* ≤ 0.001), whereas in milliseconds was shorter than the parasternal intercostal and scalene only (*p* ≤ 0.01). Likewise, time from EMG, onset to peak pressure was consistent during ITL for all muscles (*p* = 0.99) but late for the diaphragm compared to the parasternal intercostal, scalene, and sternomastoid (*p* = 0.001).

**Figure 9:**
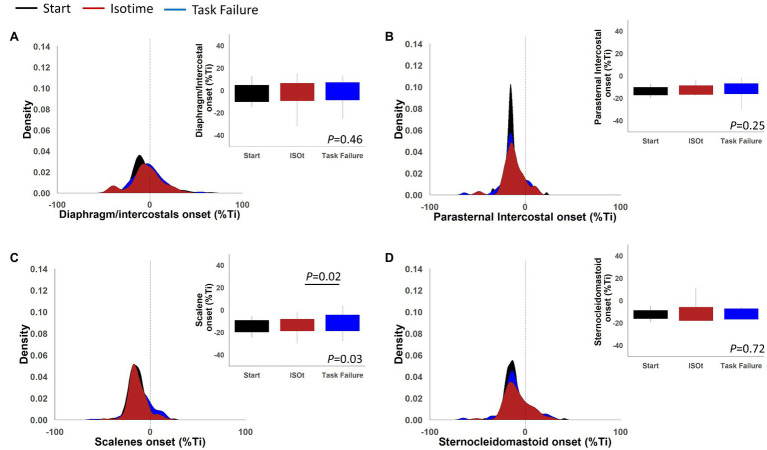
Density plots and boxplots for onset of the electromyography activity in percentage of the inspiratory time of the diaphragm/intercostal **(A)**, parasternal intercostal **(B)**, scalene **(C),** and sternocleidomastoid **(D)**.

Figure 9 shows the density plots and boxplots for the EMG, onset normalized by the duration of the inspiratory time of the diaphragm/intercostal, scalene, parasternal intercostal, and sternocleidomastoid during the ITL start, isotime, and task failure. The diaphragm/intercostal had a lower peak density than the scalene, parasternal intercostal, and sternocleidomastoid during the ITL start, isotime, and task failure, whereas the highest peak density is visualized during ITL start for the parasternal intercostal. Boxplots show there was no change in the EMG, onset variability for the diaphragm/intercostal, parasternal intercostal and sternocleidomastoid during ITL start, isotime and task failure (*p* = 0.46, *p* = 0.25, and *p* = 0.72, respectively). The scalene EMG, onset variability was greater at task failure compared to isotime (*p* = 0.02).

### Correlation Between Peak Inspiratory Flow and *Pm* With the Timing of EMG Activity

Peak inspiratory flow was significantly and inversely associated with the EMG, onset in milliseconds and normalized by the duration of the inspiratory time for the diaphragm/intercostal (*r* = −0.23 and − 0.28), scalene (*r* = − 0.50, and − 0.38), parasternal intercostal (*r* = − 0.45 and − 0.36) and sternocleidomastoid (*r* = −0.49 and − 0.42; *p* < 0.001 for all). Likewise*, Pm* was significantly and inversely associated with the EMG, onset in milliseconds and normalized by the duration of the inspiratory time for the diaphragm/intercostal (*r* = −0.20 and − 0.27), scalene (*r* = −0.30 and − 0.30), parasternal intercostal (*r* = −0.30 and − 0.32) and sternocleidomastoid (*r* = −0.27 and − 0.38; *p* < 0.001 for all). EMG, RMS and the EMG, onset in milliseconds and normalized by the duration of the inspiratory time were significantly and inversely associated for the diaphragm/intercostal (*r* = −0.25 and − 0.21), scalene (*r* = −0.16 and − 0.16), parasternal intercostal (*r* = −0.30 and − 0.29) and sternocleidomastoid (*r* = −0.26 and − 0.18; *p* < 0.001 for all). The duration of activation normalized by the duration of the inspiratory time was significantly and directly correlated with flow for the diaphragm/intercostal (*r* = 0.27), scalene (*r* = 0.43), parasternal intercostal (*r* = 0.34) and sternocleidomastoid (*r* = 0.45; *p* < 0.001 for all). However, the duration of activation in milliseconds was significantly and negatively correlated with flow for the diaphragm/intercostal (*r* = −0.50), scalene (*r* = −0.58), parasternal intercostal (*r* = −0.62) and sternocleidomastoid (*r* = −0.45; *p* < 0.001 for all). The inspiratory time was significantly and negatively correlated with inspiratory flow (*r* = −0.58; *p* < 0.001). Supplementary Figure S8 shows four scatter plots as examples of the correlations described above.

## Discussion

We developed and validated a semi-automated algorithm to detect the onset, offset, and duration of several respiratory muscles’ EMG activity with millisecond accuracy. The algorithm was an extension of a previous algorithm that filtered out ECG artifacts from the EMG of the respiratory muscles. The algorithm had good to excellent reliability compared to the manual detection performed by two different assessors. The EMG, onset detection by a semi-automated algorithm was at least 66 times faster per participant than the manual detection; it required a total of 7 min for 7 participants compared to 7.7 to 20 h for the manual assessments. Additionally, we used the algorithm to explore differences in the timing of the diaphragm/intercostal, scalene, parasternal intercostal, and sternocleidomastoid EMG, onset, EMG, offset, and duration of activation during inspiratory loading, as well as to explore whether their timing activation would be disrupted close to task failure. An ITL trial at 50% of the maximum inspiratory pressure (MIP) performed up to task failure did not change the timing of their EMG, onset, EMG offset, or duration of activation in this small sample of healthy adults (*n* = 7). To the best of our knowledge, this is the first time it is shown that the timing of the respiratory muscle EMG activity minimally affected by increased inspiratory load, despite a significant increase in breathless and the inability of the participants to overcome that load further. However, the EMG, onset of the diaphragm/intercostal occurred later and its duration of activation was shorter compared to the scalene, parasternal intercostal, and sternocleidomastoid throughout the ITL. The timing of the EMG, onset had statistically significant but small to medium correlations with peak inspiratory flow and respiratory muscle effort (*Pm*) and the amplitude of the EMG activity (RMS) for the diaphragm/intercostal, scalene, parasternal intercostal, and sternocleidomastoid.

The algorithm validity with the manual detection performed by both assessors ranged from “moderate” to “excellent.” Reliability between assessor 1 and 2 was “good.” Despite the instructions being similar to both assessors, the manual detection relies on each assessor’s interpretation of “a visual increase” on the EMG signal. Such interpretation is influenced by factors related to the quality of the signal, how much increase in the EMG signal each assessor characterize to suffice a “visual increase,” the visual acuity of the assessor, as well as other aspects such as the size of the screen available for performing the analysis. The reliability between assessor 1 and 2 was classified as “good” for all muscles (Figure 4; Supplementary Figures S1, S2) but there was great breath-by-breath variability as revealed by the upper and lower limits of agreement from the Bland-Altman plots (Figure 4; Supplementary Figures S1–S6). We have previously shown that the interrater variability in analyzing the EMG signal, for instance while analyzing the EMG RMS, can influence estimates of effects size in interventional studies despite two assessors achieving an “excellent” interrater reliability (Dacha et al., 2019). The algorithm, therefore, removes an inherent limitation of the manual analysis, that is the assessor’s subjectivity, while significantly improving time efficiency. Nevertheless, the algorithm relies in an *a priori* established threshold to determine the EMG, onset. Whether the 5% threshold used herein is the optimal threshold to estimate the timepoint of the EMG, onset dependent on the experimental protocol. However, the algorithm will consistently apply the same standardized criteria to all muscles during breath-by-breath analysis limiting potential inherent bias of manual assessments.

The timing of the EMG, onset, EMG, offset, and the duration of activation was consistent throughout ITL to task failure for all muscles. Overall, the EMG, onset of the respiratory muscles started before the beginning of the inspiration independently if based on the flow or pressure signal; however, this was different for the diaphragm/intercostals. The diaphragm/intercostals were activated significantly later (Figures 7A,D, 8A,D) and had greater variability in the timing of the EMG, onset (Figure 9), and its activity duration was shorter than the scalene, parasternal intercostal, and sternocleidomastoid (Figures 7C,F, 8C,F) even in this small sample of participants. On the other hand, previous studies have described that during quiet breathing the costal diaphragm is activated earlier than the 2nd parasternal intercostal and scalene when measurements were evaluated with needle EMG and the EMG, onset was detected visually based on the beginning of the modulation of the EMG signal (Saboisky et al., 1985; Hudson et al., 2011). These discordant results can be due to the different protocols used (i.e., quiet breathing vs. ITL), the methods used to measure muscle electrical activity (e.g., needle vs. surface EMG), and/or how the EMG, onset was detected (e.g., beginning of the modulation of the EMG signal vs. an 5% increase in the baseline EMG signal).

During quiet breathing, coordinated activity of the obligatory muscles of inspiration (i.e., diaphragm, external and parasternal intercostals, and scalene) is essential (De Troyer and Estenne, 1984; De Troyer et al., 2005; De Troyer and Boriek, 2011). Their coordinated activity allows the chest wall to move outward, decreasing intrathoracic/pleural pressure, increasing transpulmonary pressure, and ultimately generating inspiratory flow. Different stimuli will lead to changes in the way the respiratory centers activate the respiratory muscles, (De Troyer and Estenne, 1984; De Troyer and Wilson, 1985; De Troyer et al., 1998, 2005; Butler, 2007; De Troyer and Boriek, 2011; Rodrigues et al., 2019) and the respiratory center may use different strategies to meet its new demands, namely increasing the activation intensity to one or multiple obligatory muscles or activating accessory muscles of inspiration (e.g., sternocleidomastoid). The output to each specific muscle is regulated based on the muscle’s mechanical advantage (De Troyer et al., 1998; De Troyer and Boriek, 2011; Hudson et al., 2019) which depends on its morphology and length-tension relationship, and can be influenced by changes in factors such as body posture and lung volumes.

The scalene and the sternocleidomastoid are preferentially activated over the diaphragm during ITL (De Troyer and Estenne, 1984; Laghi et al., 2014; Rodrigues et al., 2019) The sternocleidomastoid has a greater proportion of type II fibers and can generate stronger and faster contractions than the diaphragm, conferring an advantage to overcome the ITL load (De Troyer and Boriek, 2011). Activating extra diaphragmatic inspiratory muscles such as scalene, parasternal intercostal, and sternocleidomastoid during ITL is a mechanism to protect the diaphragm against contractile fatigue and improve its neuromechanical coupling by limiting diaphragmatic shortening (Laghi et al., 2014). Therefore, while having a greater advantage, the scalene, parasternal intercostal, and sternocleidomastoid became the primary flow generators during ITL and are activated earlier than the diaphragm (Figure 7). Nonetheless, methodological differences cannot be ruled out. Previous studies analyzed quiet breathing, detected the EMG, onset as the time when inspiratory modulation of the EMG activity began and analyzed fewer breaths compared to ours (Saboisky et al., 1985; Hudson et al., 2011). Also, we identified the EMG, onset based on a 5% increase in the surface EMG RMS instead of determining the beginning of the inspiratory modulation from needle EMG signals.

The EMG, onset for all muscle had small to medium correlations with the flow, *Pm* and EMG RMS. *Pm* and EMG RMS did not change throughout the ITL. Changes in inspiratory flow, despite statistically significant, were small. Usually, lesser variance decreases the possibility of finding a correlation. The absence of variability in these variables may reduce the validity of these correlations. Therefore, we cannot make inferences regarding whether changes in inspiratory flow, respiratory muscle effort, or the intensity of the inspiratory drive (EMG RMS) would affect or modulate the EMG’s onset timing for the different respiratory muscles. Nevertheless, the control of the intensity and the timing activation of the respiratory muscles may be independent from each other. For instance, in a study investigating the influence of posture on the timing activation of the diaphragm and the scalene, Hudson et al. (2016) showed that the timing activation of the scalene was similar while standing or upside-down, but the amplitude of its activation decreased by about 50% in the upside-down posture. The diaphragm, however, maintained both the timing and intensity of its activation in both the seated and upside-down positions. The EMG RMS of the diaphragm/intercostal, scalene, parasternal intercostal, and sternocleidomastoid did not change throughout the ITL. *Pm* was constant and, to avoid changes in body posture to modify muscle mechanical advantage, participants were kept in the same position throughout the ITL. Hence, no changes in the EMG, RMS would be expected due to either changes in muscle effort or mechanical advantage during our protocol. Factors associated with changes in the timing of respiratory muscle EMG, onset remains to be fully elucidated.

The duration of activation did not change throughout the ITL for any muscle (Figures 7C,F, 8C,F). The duration of activation was greater than the inspiratory time for all muscle throughout the ITL (Figures 7C, 8C). The EMG, onset occurring before the beginning of inspiration would be anticipated since it is necessary to overcome the resistance applied by the threshold loading apparatus before inspiratory flow begins. The fact that the timing of the EMG, onset did not change overtime, suggest that no change in the coordination of the timing the inspiratory muscles were deployed by the respiratory centers (De Troyer and Boriek, 2011). Nevertheless, this was a cohort of healthy young adults with preserved lung function and inspiratory muscle strength. Whether this phenomenon is similar or altered in patients with impaired inspiratory muscle function (e.g., patients with diaphragm paralysis, spinal cord injury, or neuromuscular diseases) deserves further investigation.

### Strengths and Limitations

Surface EMG of the costal diaphragm is prone to crosstalk from expiratory activity of the intercostals and abdominals, which can contaminate the EMG signal (Hodges and Gandevia, 2000). Body composition and anatomy can also affect the quality of surface EMG. To minimize such influences, we only included lean individuals (sample BMI 24 ± 3 kg m^−2^) and the electrode position was adjusted to optimize the signal quality. Moreover, each subject was used as his/her own control (from ITL start to task failure) and therefore differences in body composition or anatomy would not be expected to affect the absolute microvolts measured throughout the ITL. Nevertheless, it has been shown that the timing of the EMG, onset is similar to both costal and crural diaphragm (Nguyen et al., 1985). Because previous studies have used needle EMG to detect the respiratory muscles EMG, onset and we used surface EMG, comparisons across studies may be complex. Needle EMG measures the activation of local motoneurons and represents the activity of a specific region of the muscle. We used surface EMG, which measures the superimposed signal of multiple motoneurons and represents the overall activity of the muscle region where the EMG sensor was placed (Suh et al., 2021). Nevertheless, surface EMG has been well recognized as a valid and widely used tool to study respiratory muscle function in disease and health states. Its advantages include its ease of use, and it is less risky to the patients and allows recordings to be performed during dynamic activities such as exercise. The validation of the algorithm to detect the EMG, onset included more than 2000 EMG, onset s, which, to the best of our knowledge, is considerably more than the number of EMG, onset s measured in any previous study. Additionally, we validated the algorithm by investigating its reliability with two assessors and considering four muscles. Unfortunately, we were unable to assess the timing of the EMG, onset during quiet breathing because these data were not collected during the primary study. Our sample was composed of physically fit young healthy participants, and it may not be possible to extrapolate our results to people with lung or chest wall diseases. Also, our findings were limited to one type of load, which may limit applicability to other types of load (e.g., hyperpnea). Therefore, the timing activation of the respiratory muscle EMG remains to be investigated in other populations and different types of loads.

## Conclusion

We developed and validated an algorithm to detect the onset of the EMG activity of the respiratory muscles with millisecond accuracy. The algorithm had good reliability with the manual detection performed by two independent assessors, can perform the analysis more than 60 times faster than a manual assessor, and ensures that breath-by-breath differences in the timing of the onset of the EMG activity are not biased by the assessor’s subjective interpretations. We further demonstrated that in healthy young adults (*n* = 7), an inspiratory loading task at 50% of the maximum inspiratory pressures did not disrupt the timing of the onset of the EMG activity. We propose this method may be used for objective detection of the onset of the EMG activity for the respiratory muscles.

## Data Availability Statement

The data analyzed in this study are subjected to the following licenses/restrictions: The data sets generated for this study are available on request to the corresponding author. Requests to access these data sets should be directed to antenor.rodrigues@unityhealth.to.

## Ethics Statement

The studies involving human participants were reviewed and approved by University of Toronto Health Sciences Research Ethics Board. The patients/participants provided their written informed consent to participate in this study.

## Author Contributions

AR and WR contributed to the conception and design of the study. AR, LJ, and UM contributed to data analysis. AR wrote the first draft of the manuscript. All authors contributed to the manuscript revision and read and approved the submitted version.

## Funding

This research project was funded by the Ontario Respiratory Care Society Grant #508395. AR was funded by a Fellowship from the Faculty of Medicine, University of Toronto.

## Conflict of Interest

The authors declare that the research was conducted in the absence of any commercial or financial relationships that could be construed as a potential conflict of interest.

## Publisher’s Note

All claims expressed in this article are solely those of the authors and do not necessarily represent those of their affiliated organizations, or those of the publisher, the editors and the reviewers. Any product that may be evaluated in this article, or claim that may be made by its manufacturer, is not guaranteed or endorsed by the publisher.
