# Supplementary material for: Semi-automated Detection of the Timing of Respiratory Muscle Activity: Validation and First Application
Source: Front Physiol. 2022 Jan 3;12:794598. doi: 10.3389/fphys.2021.794598 (PMC8762204; doi:10.3389/fphys.2021.794598)
Supplement: Supplementary file 1 [file Data_Sheet_1.pdf]

Supplementary Material

SEMI-AUTOMATED DETECTION OF THE TIMING OF RESPIRATORY MUSCLE ACTIVITY: VALIDATION AND FIRST APPLICATION

Antenor Rodrigues,[1*] Luc Janssens,[2,3] Daniel Langer,[3,4] Umi Matsumura,[5] Dmitry Rozenberg,[6,7] Laurent Brochard,[1,8,9] W. Darlene Reid[8,10,11]

1.1 Supplementary Figures

Figure S1. Bland-Altman plots and ICC's values for the breath-by-breath detection of *dP* of EMG,onset by assessor 1 and 2 for the diaphragm/intercostal (A), parasternal intercostal (B), scalene (C) and sternocleidomastoid (D). AVG: average bias between the results from both assessors. UL and LL: 95% confidence interval of the difference between the results from both assessors. n: number of EMG,onset analyzed. *dP*: phased difference between the onset of the electrical activity of the muscle and the start of the inspiration calculated (see text for further details).

Figure S2. Bland and Altman plots and ICC's values for the breath-by-breath detection of percentage of the inspiratory time (%Ti) of EMG,onset by assessor 1 and 2 for the diaphragm/intercostal (A), parasternal intercostal (B), scalene (C) and sternocleidomastoid (D). AVG: average bias between the results from both assessors. UL and LL: 95% confidence interval of the difference between the results from both assessors. n: number of EMG,onset analyzed(see text for further details).

Figure S3. Bland and Altman plots and ICC's values for the breath-by-breath detection of *dP* of EMG,onset by assessor 1 and the algorithm for the diaphragm/intercostal (A), parasternal intercostal (B), scalene (C) and sternocleidomastoid (D). AVG: average bias between the results from both assessors. UL and LL: 95% confidence interval of the difference between the results from both assessors. n: number of EMG,onset analyzed. *dP*: phased difference between the onset of the electrical activity of the muscle and the start of the inspiration calculated (see text for further details).

Figure S4. Bland and Altman plots and ICC's values for the breath-by-breath detection of percentage of the inspiratory time (%Ti) of EMG,onset by assessor 1 and the algorithm for the diaphragm/intercostal (A), parasternal intercostal (B), scalene (C) and sternocleidomastoid (D). AVG: average bias between

the results from both assessors. UL and LL: 95% confidence interval of the difference between the results from both assessors. n: number of EMG,onset analyzed (see text for further details).

Figure S5. Bland and Altman plots and ICC's values for the breath-by-breath detection of *dP* of EMG,onset by assessor 2 and the algorithm for the diaphragm/intercostal (A), parasternal intercostal (B), scalene (C) and sternocleidomastoid (D). AVG: average bias between the results from both assessors. UL and LL: 95% confidence interval of the difference between the results from both assessors. n: number of EMG,onset analyzed. *dP*: phased difference between the onset of the electrical activity of the muscle and the start of the inspiration calculated (see text for further details).

Figure S6. Bland and Altman plots and ICC's values for the breath-by-breath detection of percentage of the inspiratory time (%Ti) of EMG,onset by assessor 2 and the algorithm for the diaphragm/intercostal (A), parasternal intercostal (B), scalene (C) and sternocleidomastoid (D). AVG: average bias between the results from both assessors. UL and LL: 95% confidence interval of the difference between the results from both assessors. n: number of EMG,onset analyzed (see text for further details).

Figure S7. Amplitude of the muscle activation during the inspiratory threshold loading. RMS: root mean square of the electromyography. Data is depicted as mean±SE. *$P<0.001$ diaphragm/intercostals vs. parasternal intercostal, scalene and sternocleidomastoid. ¥ $P=0.016$ parasternal intercostal vs. scalene.

Figure S8. Correlations between (A) diaphragm/intercostal EMG,onset in percentage of the inspiratory time (%Ti) and diaphragm/intercostal EMG RMS, (B) diaphragm/intercostal EMG,onset in percentage of the inspiratory time (%Ti) and inspiratory flow, (C) diaphragm/intercostal EMG,onset in seconds (dP) and inspiratory flow, (D) diaphragm/intercostal duration of contraction and inspiratory flow.

Figure S1

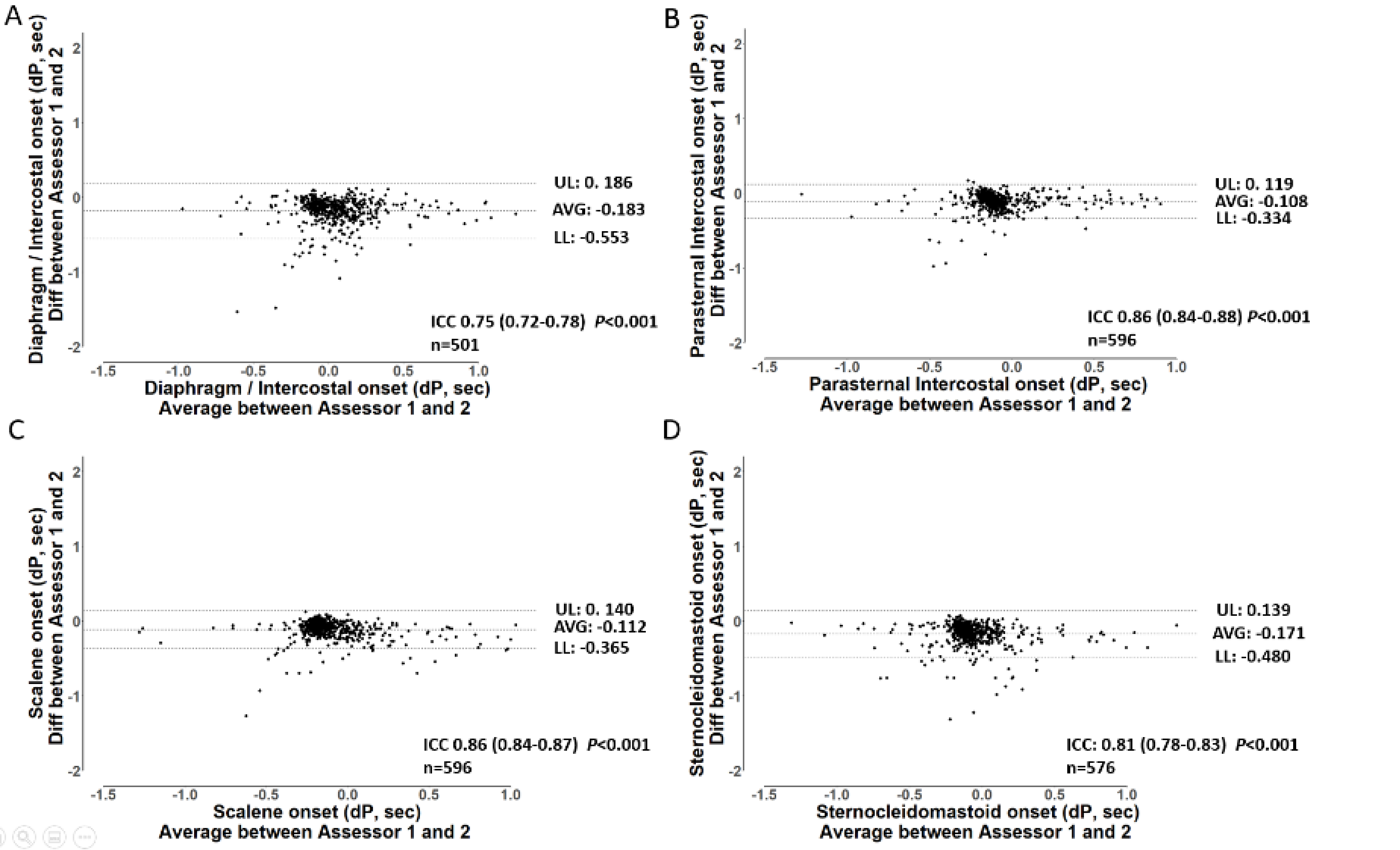
A
Diaphragm / Intercostal onset (dP, sec)
Diff between Assessor 1 and 2
UL: 0. 186
AVG: -0.183
LL: -0.553
ICC 0.75 (0.72-0.78) P<0.001
n=501
Diaphragm / Intercostal onset (dP, sec)
Average between Assessor 1 and 2
B
Parasternal Intercostal onset (dP, sec)
Diff between Assessor 1 and 2
UL: 0. 119
AVG: -0.108
LL: -0.334
ICC 0.86 (0.84-0.88) P<0.001
n=596
Parasternal Intercostal onset (dP, sec)
Average between Assessor 1 and 2
C
Scalene onset (dP, sec)
Diff between Assessor 1 and 2
UL: 0. 140
AVG: -0.112
LL: -0.365
ICC 0.86 (0.84-0.87) P<0.001
n=596
Scalene onset (dP, sec)
Average between Assessor 1 and 2
D
Sternocleidomastoid onset (dP, sec)
Diff between Assessor 1 and 2
UL: 0.139
AVG: -0.171
LL: -0.480
ICC: 0.81 (0.78-0.83) P<0.001
n=576
Sternocleidomastoid onset (dP, sec)
Average between Assessor 1 and 2

Figure S2

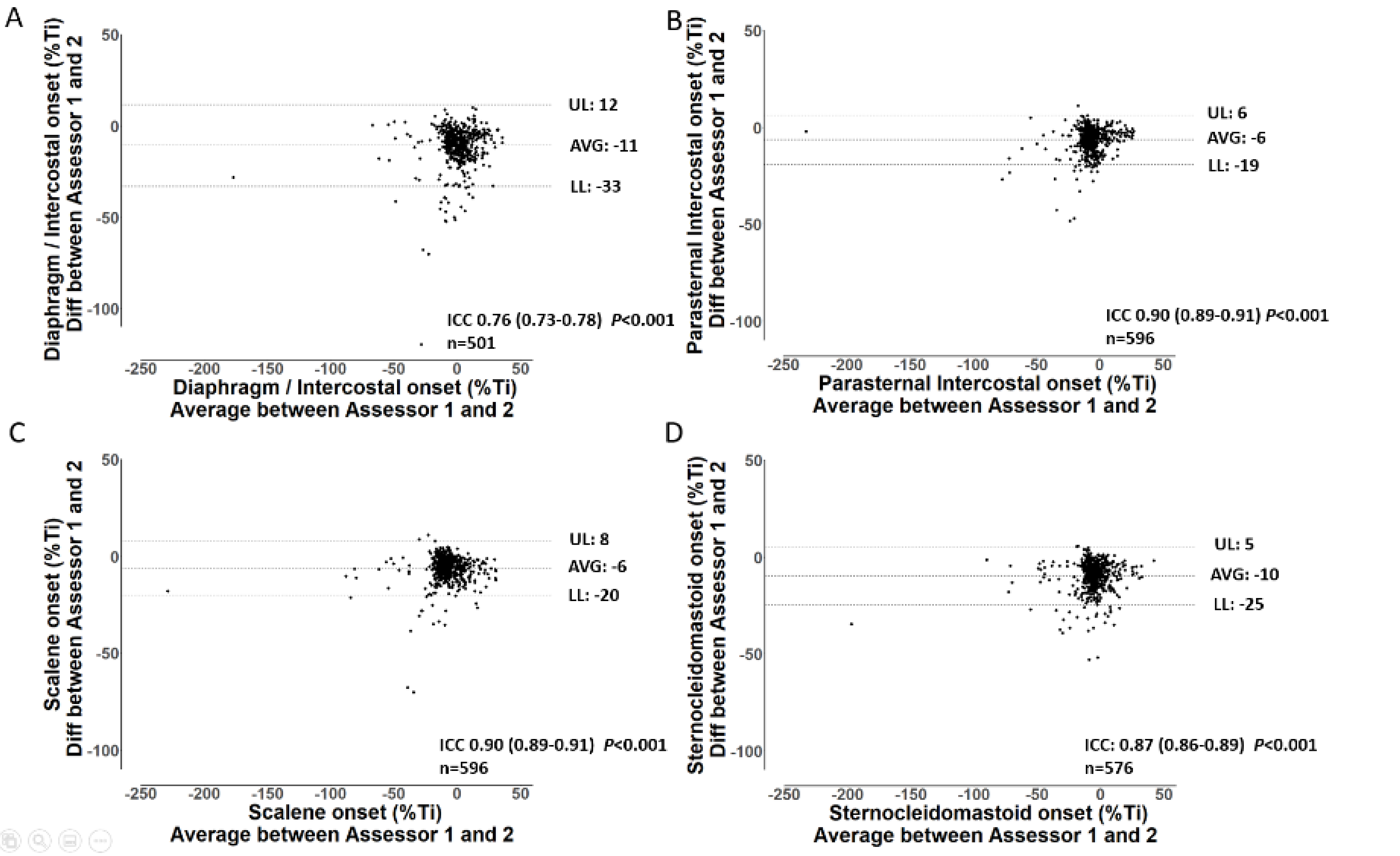
A
Diaphragm / Intercostal onset (%Ti)
Diff between Assessor 1 and 2
UL: 12
AVG: -11
LL: -33
ICC 0.76 (0.73-0.78) P<0.001
n=501
Diaphragm / Intercostal onset (%Ti)
Average between Assessor 1 and 2
B
Parasternal Intercostal onset (%Ti)
Diff between Assessor 1 and 2
UL: 6
AVG: -6
LL: -19
ICC 0.90 (0.89-0.91) P<0.001
n=596
Parasternal Intercostal onset (%Ti)
Average between Assessor 1 and 2
C
Scalene onset (%Ti)
Diff between Assessor 1 and 2
UL: 8
AVG: -6
LL: -20
ICC 0.90 (0.89-0.91) P<0.001
n=596
Scalene onset (%Ti)
Average between Assessor 1 and 2
D
Sternocleidomastoid onset (%Ti)
Diff between Assessor 1 and 2
UL: 5
AVG: -10
LL: -25
ICC: 0.87 (0.86-0.89) P<0.001
n=576
Sternocleidomastoid onset (%Ti)
Average between Assessor 1 and 2

Figure S3

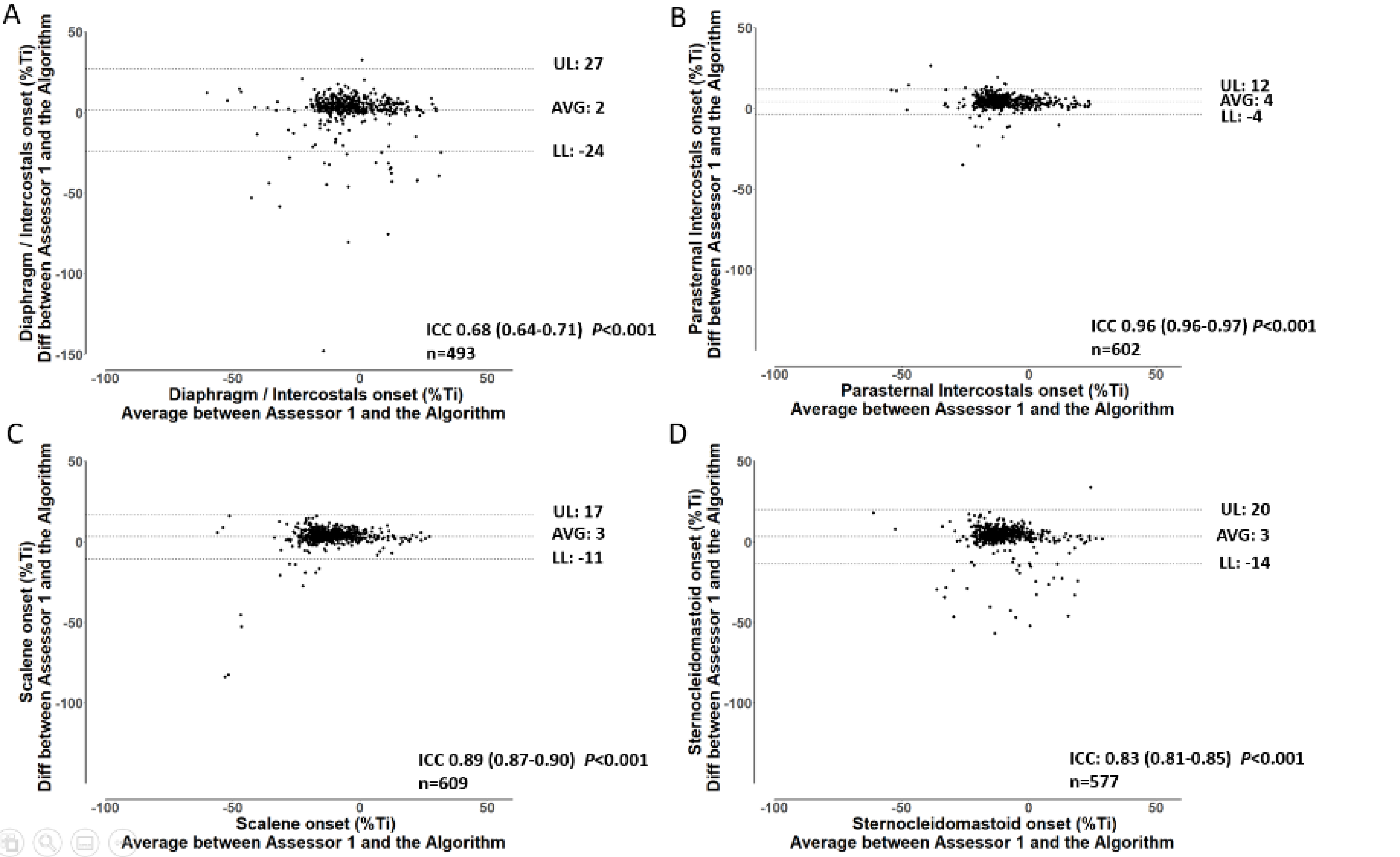
A
UL: 27
AVG: 2
LL: -24
ICC 0.68 (0.64-0.71) P<0.001
n=493
Diaphragm / Intercostals onset (%Ti)
Diff between Assessor 1 and the Algorithm
Diaphragm / Intercostals onset (%Ti)
Average between Assessor 1 and the Algorithm
B
UL: 12
AVG: 4
LL: -4
ICC 0.96 (0.96-0.97) P<0.001
n=602
Parasternal Intercostals onset (%Ti)
Diff between Assessor 1 and the Algorithm
Parasternal Intercostals onset (%Ti)
Average between Assessor 1 and the Algorithm
C
UL: 17
AVG: 3
LL: -11
ICC 0.89 (0.87-0.90) P<0.001
n=609
Scalene onset (%Ti)
Diff between Assessor 1 and the Algorithm
Scalene onset (%Ti)
Average between Assessor 1 and the Algorithm
D
UL: 20
AVG: 3
LL: -14
ICC: 0.83 (0.81-0.85) P<0.001
n=577
Sternocleidomastoid onset (%Ti)
Diff between Assessor 1 and the Algorithm
Sternocleidomastoid onset (%Ti)
Average between Assessor 1 and the Algorithm

Figure S4

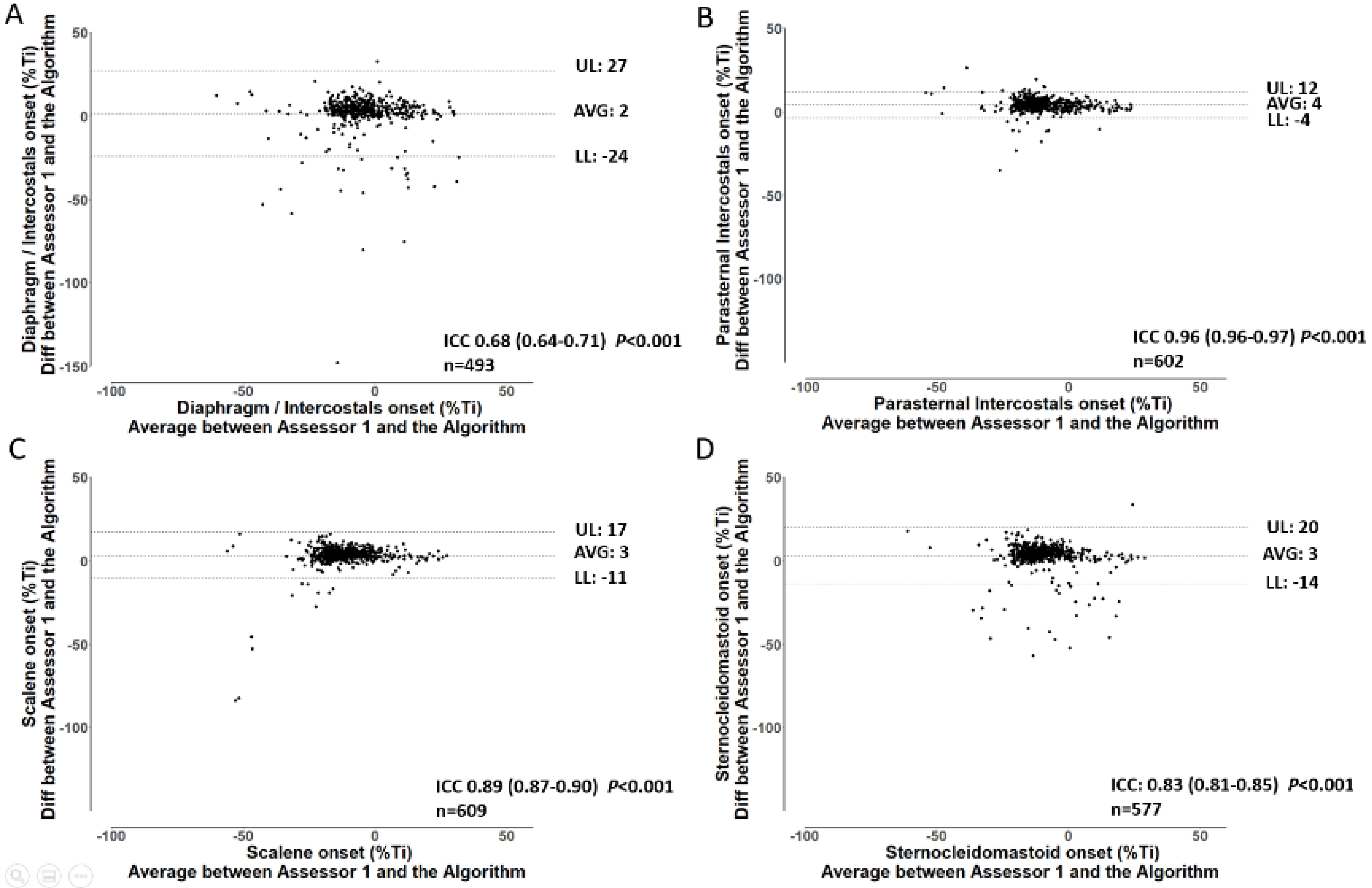
A
Diaphragm / Intercostals onset (%Ti)
Diff between Assessor 1 and the Algorithm
UL: 27
AVG: 2
LL: -24
ICC 0.68 (0.64-0.71) P<0.001
n=493
Diaphragm / Intercostals onset (%Ti)
Average between Assessor 1 and the Algorithm
B
Parasternal Intercostals onset (%Ti)
Diff between Assessor 1 and the Algorithm
UL: 12
AVG: 4
LL: -4
ICC 0.96 (0.96-0.97) P<0.001
n=602
Parasternal Intercostals onset (%Ti)
Average between Assessor 1 and the Algorithm
C
Scalene onset (%Ti)
Diff between Assessor 1 and the Algorithm
UL: 17
AVG: 3
LL: -11
ICC 0.89 (0.87-0.90) P<0.001
n=609
Scalene onset (%Ti)
Average between Assessor 1 and the Algorithm
D
Sternocleidomastoid onset (%Ti)
Diff between Assessor 1 and the Algorithm
UL: 20
AVG: 3
LL: -14
ICC: 0.83 (0.81-0.85) P<0.001
n=577
Sternocleidomastoid onset (%Ti)
Average between Assessor 1 and the Algorithm

Figure S5

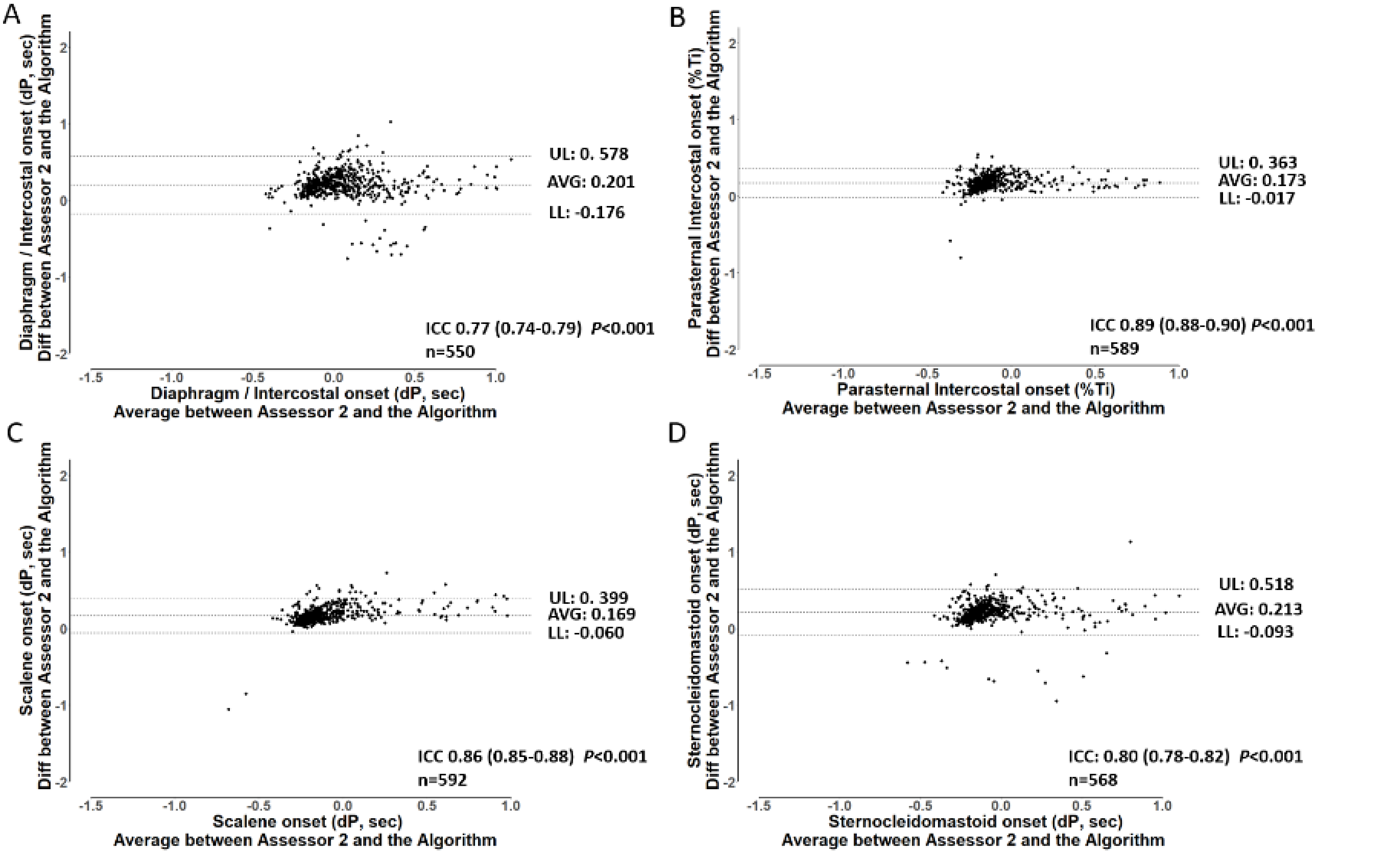
A
Diaphragm / Intercostal onset (dP, sec)
Diff between Assessor 2 and the Algorithm
UL: 0. 578
AVG: 0.201
LL: -0.176
ICC 0.77 (0.74-0.79) P<0.001
n=550
Diaphragm / Intercostal onset (dP, sec)
Average between Assessor 2 and the Algorithm
B
Parasternal Intercostal onset (%Ti)
Diff between Assessor 2 and the Algorithm
UL: 0. 363
AVG: 0.173
LL: -0.017
ICC 0.89 (0.88-0.90) P<0.001
n=589
Parasternal Intercostal onset (%Ti)
Average between Assessor 2 and the Algorithm
C
Scalene onset (dP, sec)
Diff between Assessor 2 and the Algorithm
UL: 0. 399
AVG: 0.169
LL: -0.060
ICC 0.86 (0.85-0.88) P<0.001
n=592
Scalene onset (dP, sec)
Average between Assessor 2 and the Algorithm
D
Sternocleidomastoid onset (dP, sec)
Diff between Assessor 2 and the Algorithm
UL: 0.518
AVG: 0.213
LL: -0.093
ICC: 0.80 (0.78-0.82) P<0.001
n=568
Sternocleidomastoid onset (dP, sec)
Average between Assessor 2 and the Algorithm

Figure S6

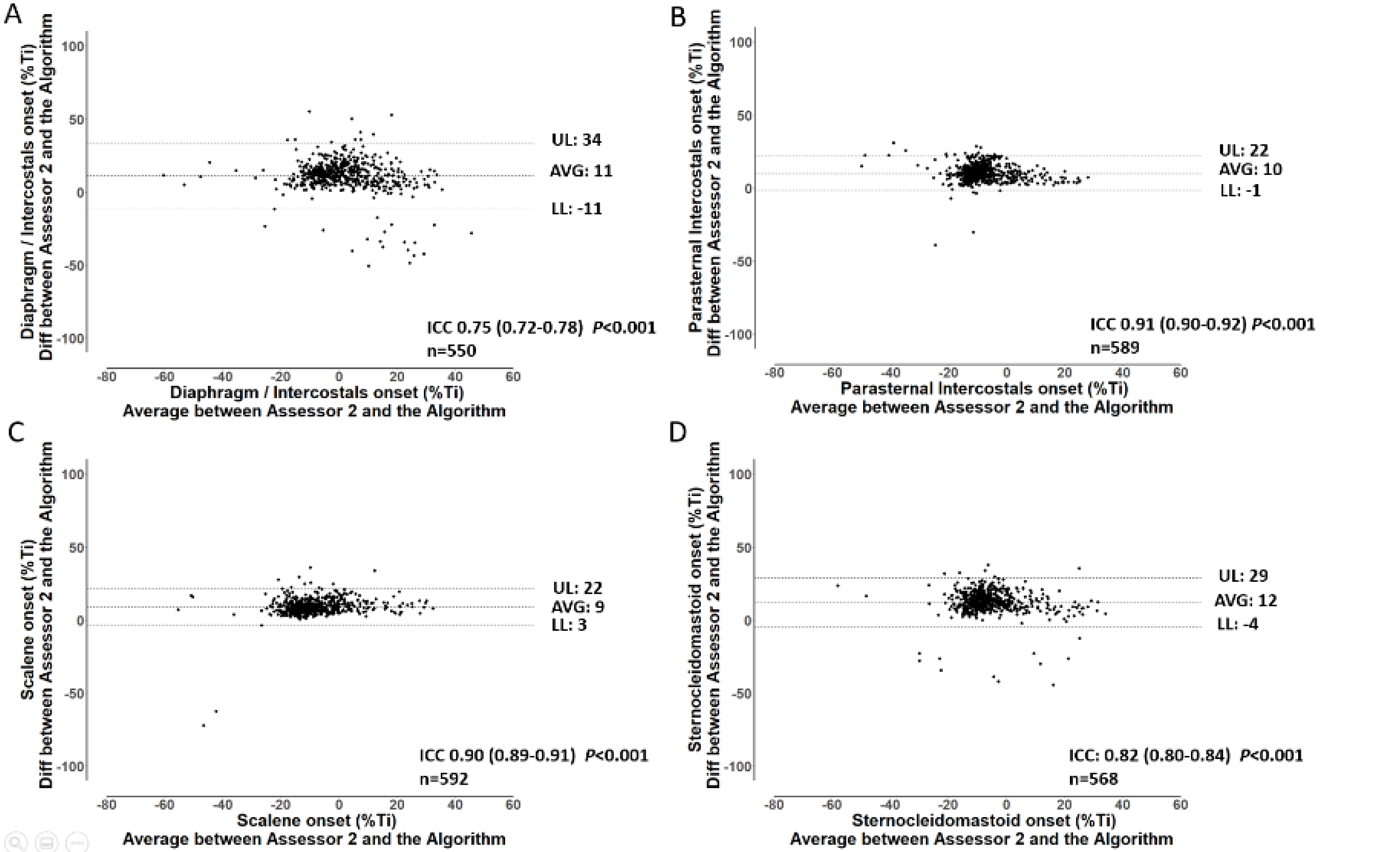
A
Diaphragm / Intercostals onset (%Ti)
Diff between Assessor 2 and the Algorithm
UL: 34
AVG: 11
LL: -11
ICC 0.75 (0.72-0.78) P<0.001
n=550
Diaphragm / Intercostals onset (%Ti)
Average between Assessor 2 and the Algorithm
B
Parasternal Intercostals onset (%Ti)
Diff between Assessor 2 and the Algorithm
UL: 22
AVG: 10
LL: -1
ICC 0.91 (0.90-0.92) P<0.001
n=589
Parasternal Intercostals onset (%Ti)
Average between Assessor 2 and the Algorithm
C
Scalene onset (%Ti)
Diff between Assessor 2 and the Algorithm
UL: 22
AVG: 9
LL: 3
ICC 0.90 (0.89-0.91) P<0.001
n=592
Scalene onset (%Ti)
Average between Assessor 2 and the Algorithm
D
Sternocleidomastoid onset (%Ti)
Diff between Assessor 2 and the Algorithm
UL: 29
AVG: 12
LL: -4
ICC: 0.82 (0.80-0.84) P<0.001
n=568
Sternocleidomastoid onset (%Ti)
Average between Assessor 2 and the Algorithm

Figure S7

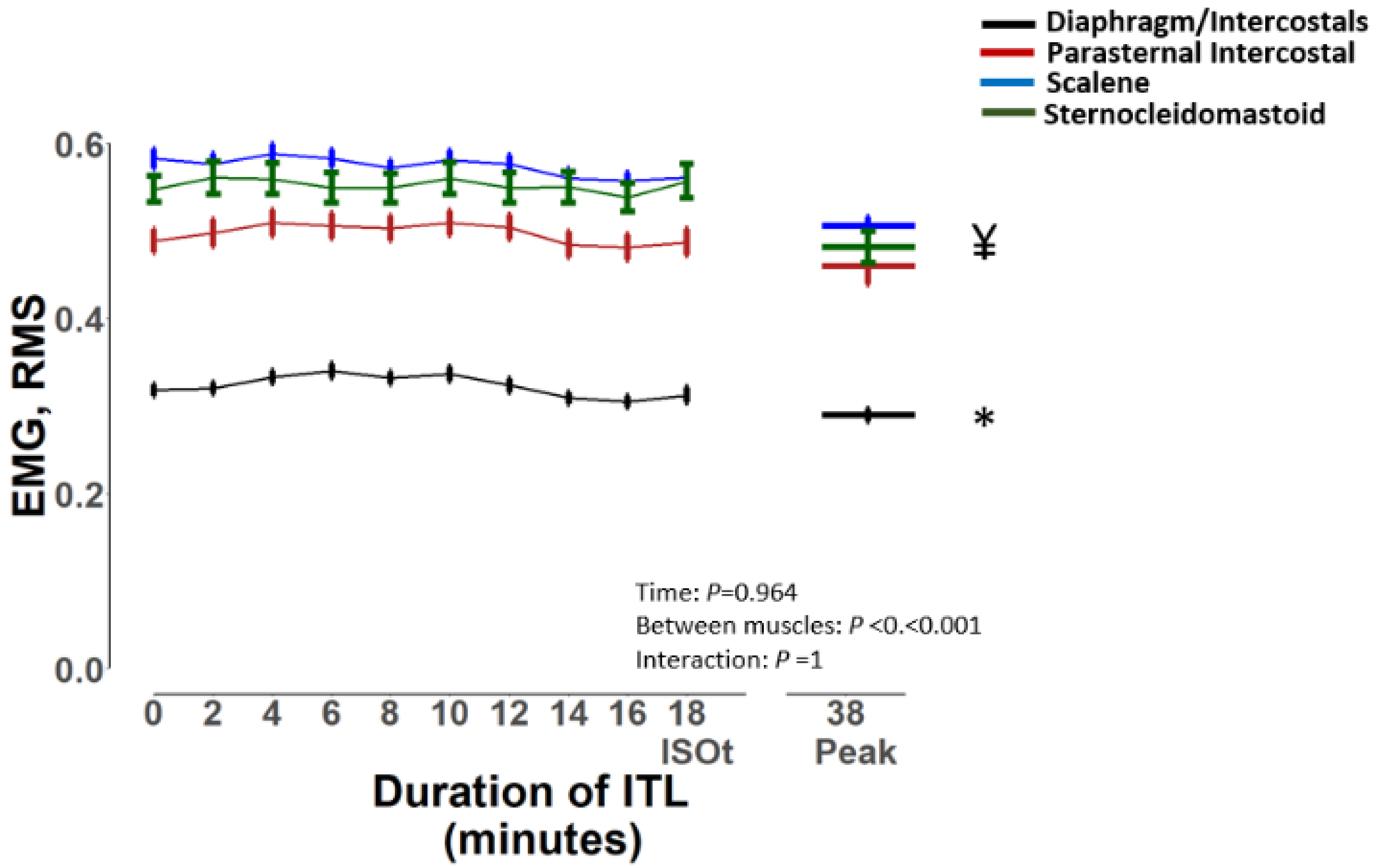
Diaphragm/Intercostals
Parasternal Intercostal
Scalene
Sternocleidomastoid
EMG, RMS
0.6
0.4
0.2
0.0
¥
*
Time: P=0.964
Between muscles: P <0.<0.001
Interaction: P =1
0 2 4 6 8 10 12 14 16 18
ISOt
38
Peak
Duration of ITL
(minutes)

Figure S8

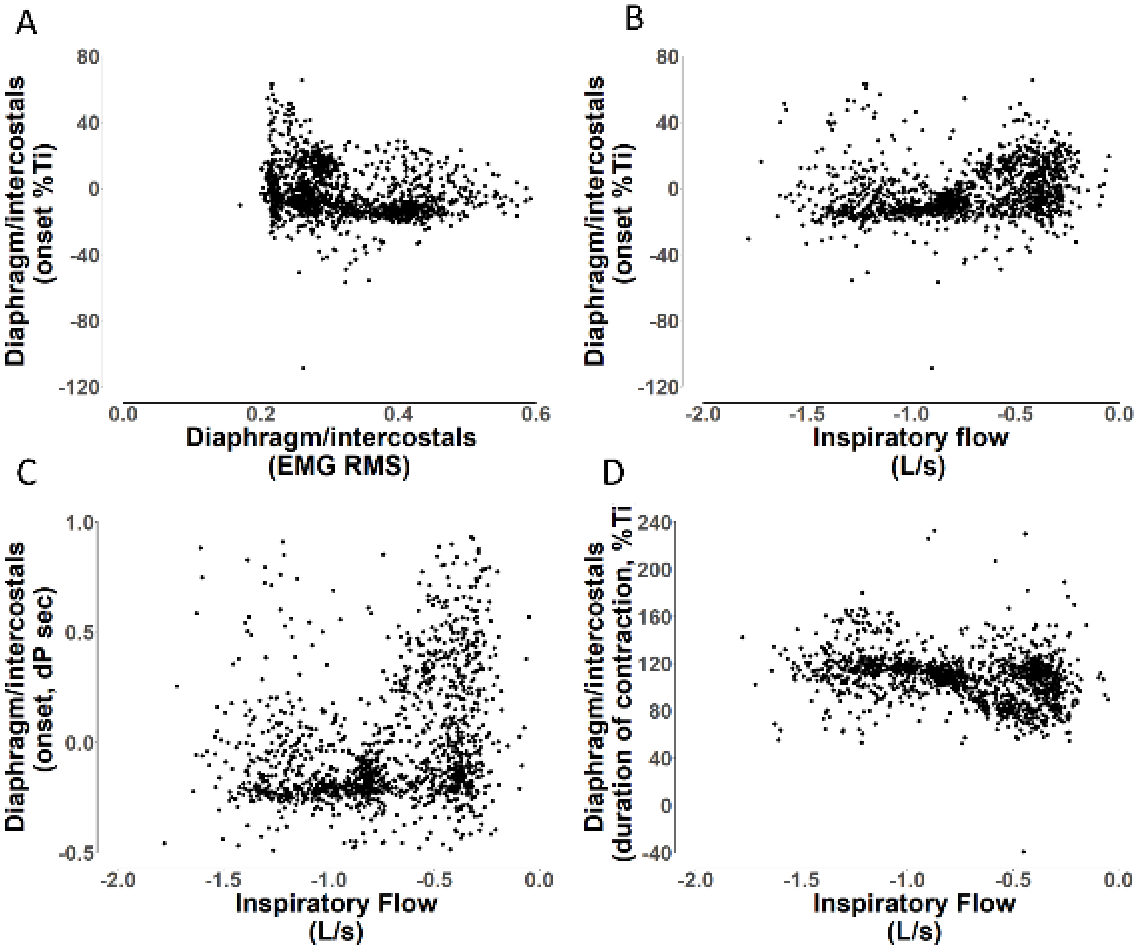
A
B
C
D
Diaphragm/intercostals (onset %Ti)
Diaphragm/intercostals (EMG RMS)
Inspiratory flow (L/s)
Diaphragm/intercostals (onset, dP sec)
Inspiratory Flow (L/s)
Diaphragm/intercostals (duration of contraction, %Ti)
80
40
0
-40
-80
-120
0.0
0.2
0.4
0.6
-2.0
-1.5
-1.0
-0.5
1.0
0.5
240
200
160
120
